# Loss of the RNA polymerase III repressor MAF1 confers obesity resistance

**DOI:** 10.1101/gad.258350.115

**Published:** 2015-05-01

**Authors:** Nicolas Bonhoure, Ashlee Byrnes, Robyn D. Moir, Wassim Hodroj, Frédéric Preitner, Viviane Praz, Genevieve Marcelin, Streamson C. Chua, Nuria Martinez-Lopez, Rajat Singh, Norman Moullan, Johan Auwerx, Gilles Willemin, Hardik Shah, Kirsten Hartil, Bhavapriya Vaitheesvaran, Irwin Kurland, Nouria Hernandez, Ian M. Willis

**Affiliations:** 1 Center for Integrative Genomics, Faculty of Biology and Medicine, University of Lausanne, 1015 Lausanne, Switzerland;; 2 Department of Biochemistry, Albert Einstein College of Medicine, Bronx, New York 10461, USA;; 3 Mouse Metabolic Evaluation Facility, Center for Integrative Genomics, University of Lausanne, 1015 Lausanne, Switzerland;; 4 Swiss Institute of Bioinformatics, 1015 Lausanne, Switzerland;; 5 Division of Endocrinology, Department of Medicine, Albert Einstein College of Medicine, Bronx, New York 10461, USA;; 6 Diabetes Research Center, Albert Einstein College of Medicine, Bronx, New York 10461, USA;; 7 Laboratory for Integrative and Systems Physiology, Ecole Polytechnique Fédérale de Lausanne (EPFL), 1015 Lausanne, Switzerland

**Keywords:** obesity, RNA polymerase III, metabolic efficiency, MAF1, autophagy, futile cycling, polyamines

## Abstract

MAF1 is a global repressor of RNA polymerase III transcription that regulates the expression of highly abundant noncoding RNAs in response to nutrient availability and cellular stress. Thus, MAF1 function is thought to be important for metabolic economy. Here we show that a whole-body knockout of *Maf1* in mice confers resistance to diet-induced obesity and nonalcoholic fatty liver disease by reducing food intake and increasing metabolic inefficiency. Energy expenditure in *Maf1*^−/−^ mice is increased by several mechanisms. Precursor tRNA synthesis was increased in multiple tissues without significant effects on mature tRNA levels, implying increased turnover in a futile tRNA cycle. Elevated futile cycling of hepatic lipids was also observed. Metabolite profiling of the liver and skeletal muscle revealed elevated levels of many amino acids and spermidine, which links the induction of autophagy in *Maf1*^−/−^ mice with their extended life span. The increase in spermidine was accompanied by reduced levels of nicotinamide *N*-methyltransferase, which promotes polyamine synthesis, enables nicotinamide salvage to regenerate NAD^+^, and is associated with obesity resistance. Consistent with this, NAD^+^ levels were increased in muscle. The importance of MAF1 for metabolic economy reveals the potential for MAF1 modulators to protect against obesity and its harmful consequences.

## Abstract (precis)

MAF1 is a global repressor of RNA polymerase III transcription that regulates the expression of highly abundant noncoding RNAs in response to nutrient availability and cellular stress. Bonhoure et al. show that a whole-body knockout of Maf1 in mice confers resistance to diet-induced obesity and nonalcoholic fatty liver disease by reducing food intake and increasing energy expenditure by several mechanisms.

In natural populations, metabolic efficiency promotes survival in stressful environments, such as when the quality or quantity of food is limited (Parsons 2007). However, as evidenced by the global obesity epidemic and its associated comorbidities (e.g., insulin resistance, type 2 diabetes, cardiovascular disease, nonalcoholic fatty liver disease, and cancer) (Guh et al. 2009; Flegal et al. 2010; Unger and Scherer 2010), metabolic efficiency has become a liability for a large number of modern day humans. Reducing obesity through diet and exercise produces health benefits, but maintaining weight loss over the long term remains a challenge for most overweight people (Kraschnewski et al. 2010; Maclean et al. 2011), and pharmacological approaches to reduce food intake or absorption have undesirable side effects or safety concerns (Tseng et al. 2010; Clapham and Arch 2011). With the identification of functional brown adipose tissue (BAT) in adult humans and the inducible browning of white adipose tissue (WAT), new strategies to increase energy expenditure have emerged as promising therapies for obesity and metabolic disease (Harms and Seale 2013; Rosen and Spiegelman 2014). These approaches stimulate facultative thermogenic responses that uncouple substrate oxidation from ATP synthesis, dissipate the mitochondrial proton gradient, and release chemical energy as heat. Other possibilities to enhance energy expenditure by decreasing the metabolic efficiency of obligatory cellular processes remain largely unexplored (Alekseev et al. 2010; Anunciado-Koza et al. 2011; Oie et al. 2014).

Ribosome biogenesis has long been recognized as a significant consumer of metabolic energy, with ∼60% of the nucleotides polymerized in nuclear gene transcription of exponentially growing cells going toward the synthesis of the large ribosomal RNAs (rRNAs) (Warner 1999; Grummt 2013). The energetic cost of this synthesis along with the production of 5S rRNA and tRNAs underlies a biological imperative for tight control of these processes in all organisms. Thus, metabolic economy is ensured when nutrients are limiting and under various stress conditions by regulatory systems that rapidly repress transcription involving the protein synthetic machinery (Warner 1999; Grummt 2013; Moir and Willis 2013). In higher eukaryotes, repression of rDNA transcription in response to nutrient deprivation is mediated in part by the energy-dependent nucleolar silencing complex (eNoSC). eNoSC binding throughout the rDNA repeat is achieved via the nucleolar protein nucleomethylin (NML) and its interaction with histone H3 dimethylated at Lys9. Together with the action of other eNoSC subunits, a repressive chromatin structure is established by the SIRT1 histone deacetylase and the histone H3 methyltransferase Suv39h1, leading to the repression of rDNA transcription (Murayama et al. 2008). The biological importance of the energy conservation provided by this repression is indicated by the resistance of mice with a liver-specific knockout of NML to diet-induced obesity (Oie et al. 2014). In the absence of NML, increased rDNA transcription in high-fat-fed mice promotes hepatic energy expenditure and alters lipid metabolism, leading to reduced fat accumulation and reduced body weight gain. Thus, repression of hepatic rDNA transcription allows excess energy storage as fat.

MAF1 functions to promote metabolic economy by repressing RNA polymerase III (Pol III) transcription of highly abundant cellular RNAs under conditions of nutrient limitation and cellular stress (Upadhya et al. 2002; Reina et al. 2006). In yeast, MAF1 is required universally for this response, and, in its absence, strains exhibit reduced fitness, stress sensitivity, altered respiratory metabolism, and decreased sporulation efficiency (Cherry et al. 2012). These phenotypes can be rationalized by the inappropriate diversion of metabolic resources into the energetically costly synthesis of 5S RNA and tRNAs, which together account for ∼15% of total RNA. MAF1 is a terminal node in the target of rapamycin (TOR) signaling network, which drives cell growth, controls metabolism, and contributes to metabolic disease, cancer, and aging (Michels et al. 2010; Zoncu et al. 2011; Moir and Willis 2013). The phospho-regulation of MAF1, its interactions with the RNA Pol III transcription machinery, and its function in transcriptional repression are conserved from yeast to mammals, but the impact of its ablation has not been assessed in metazoans.

## Results

### Obesity and fatty liver resistance of *Maf1*^−/−^ mice

**Figure 1.**
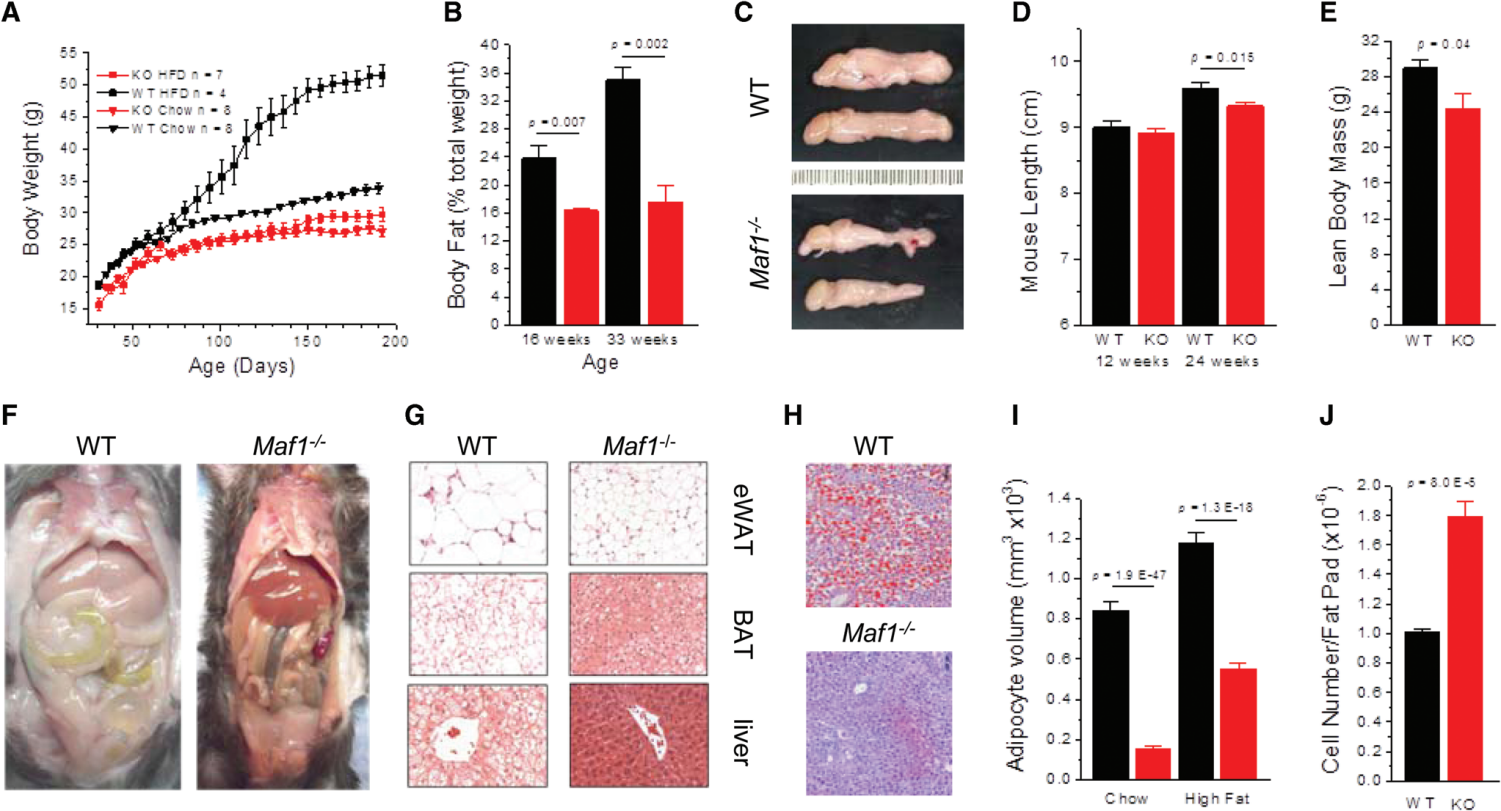
*Maf1*^−/−^ mice exhibit resistance to diet-induced obesity and fatty liver disease. (*A*) Body weight curves of wild-type (WT) and *Maf1*^−/−^ (KO) animals on chow diets and HFDs. (*B*) Fat mass as a percentage body weight for chow-fed mice at 16 and 33 wk (*n* = 3 per group). (*C*) Epididymal fat pads harvested from chow-fed wild-type and *Maf1*^−/−^ mice at 5 mo of age. Images are oriented with the testes to the *left*. (*D*) Nose to anus body length of chow-fed mice (*n* = 8 per group at 3 mo; *n* = 19 per group at 6 mo of age). (*E*) Lean body mass of chow-fed mice at 12 mo of age (*n* = 5 wild type; *n* = 4 knockout). (*F*) Gross pathology of 7-mo-old HFD-fed mice (representative of three animals per group). (*G*) Hematoxylin and eosin (H&E)-stained eWAT, BAT, and livers from the HFD-fed mice in *F*. Images are at the same magnification. (*H*) Oil-Red-O staining of livers from 12-mo-old chow-fed mice. (*I*) Estimation of adipocyte cell volumes for mice on chow-fed diets versus HFDs (see also Supplemental Fig. S1G). (*J*) eWAT fat pad cell counts for 12-mo-old chow-fed mice (*n* = 5 per group). (Black) Wild-type; (red) *Maf1*^−/−^. All values are presented as the mean ± SEM.

MAF1 is encoded by a single ubiquitously expressed gene in mice and humans (Wu et al. 2009) and was knocked out in mouse embryonic stem cells by homologous recombination (Supplemental Fig. S1A–C). Crosses of *Maf1*^+/−^ mice generated in the C57Bl/6J background produced *Maf1*^−/−^ progeny in numbers that were not statistically different from Mendelian expectation (Supplemental Fig. S1D). Thus, the whole-body knockout is unconditionally viable. Interbreeding of *Maf1*^−/−^ animals revealed reduced fertility and fecundity compared with wild-type mice (Supplemental Fig. S1E). *Maf1*^−/−^ mice appeared normal at birth but exhibited lower body weight compared with age-matched controls after weaning (Fig. 1A; Supplemental Fig. S1F). These differences were extreme under a high-fat diet (HFD), as wild-type mice rapidly became obese. In contrast, *Maf1*^−/−^ mice maintained essentially the same body weight on HFDs and regular chow diets (Fig. 1A). Body composition analyses showed that chow-fed *Maf1*^−/−^ mice had substantially less fat as a percentage of total body weight compared with wild-type mice, consistent with the reduced size of their epididymal fat pads (Fig. 1B,C). In older animals (>6 mo of age), differences in body length and absolute lean body mass were also apparent, suggesting an overall reduction in the growth rate of the knockout (Fig. 1D,E). *Maf1*^−/−^ mice maintained on a HFD had little omental and subcutaneous adipose tissue compared with controls (Fig. 1F). In contrast, epididymal WAT (eWAT) and BAT were retained in the knockout, yet the adipocytes did not become hypertrophic as seen in wild-type tissue (Fig. 1G). eWAT adipocyte cell volume was markedly reduced in *Maf1*^−/−^ mice on both low-fat diets and HFDs (Fig. 1G,I; Supplemental Fig. S1G). These differences in cell volume (especially for chow-fed mice) predict larger differences in epididymal fat pad size than were observed (Fig. 1C). However, *Maf1*^−/−^ mice had almost twice the number of adipocytes in this fat depot as wild-type mice (Fig. 1J). Wild-type mice fed a HFD for 6 mo and chow-fed mice at 1 yr exhibited severe hepatocellular swelling due to lipid droplet (LD) accumulation in the liver. This defining phenotype of nonalcoholic fatty liver disease was not observed in the knockout (Fig. 1F–H; Supplemental Fig. S1G). To confirm that the body weight phenotype of *Maf1*^−/−^ mice was due to the loss of the MAF1 protein and was not an effect of the deletion on expression of some other gene, *Maf1*-null mice were generated with targeted zinc finger nucleases. Two different C57Bl/6J lines were obtained: one with a single-base-pair insertion and another with an 8-base-pair (bp) deletion. Both mutations change the reading frame after Thr64, resulting in translation termination shortly thereafter (Supplemental Fig. S2A). Under ad libitum high-fat feeding, these *Maf1*-null mice showed a dramatic resistance to weight gain similar to that of mice lacking the entire coding region (Fig. 1A; Supplemental Fig. S2B). We conclude that whole body loss of MAF1 results in mice that are lean and profoundly resistant to diet-induced obesity and fatty liver disease. All subsequent experiments in this study were performed with the complete gene knockout.

### Reduced food intake and metabolic inefficiency

**Figure 2.**
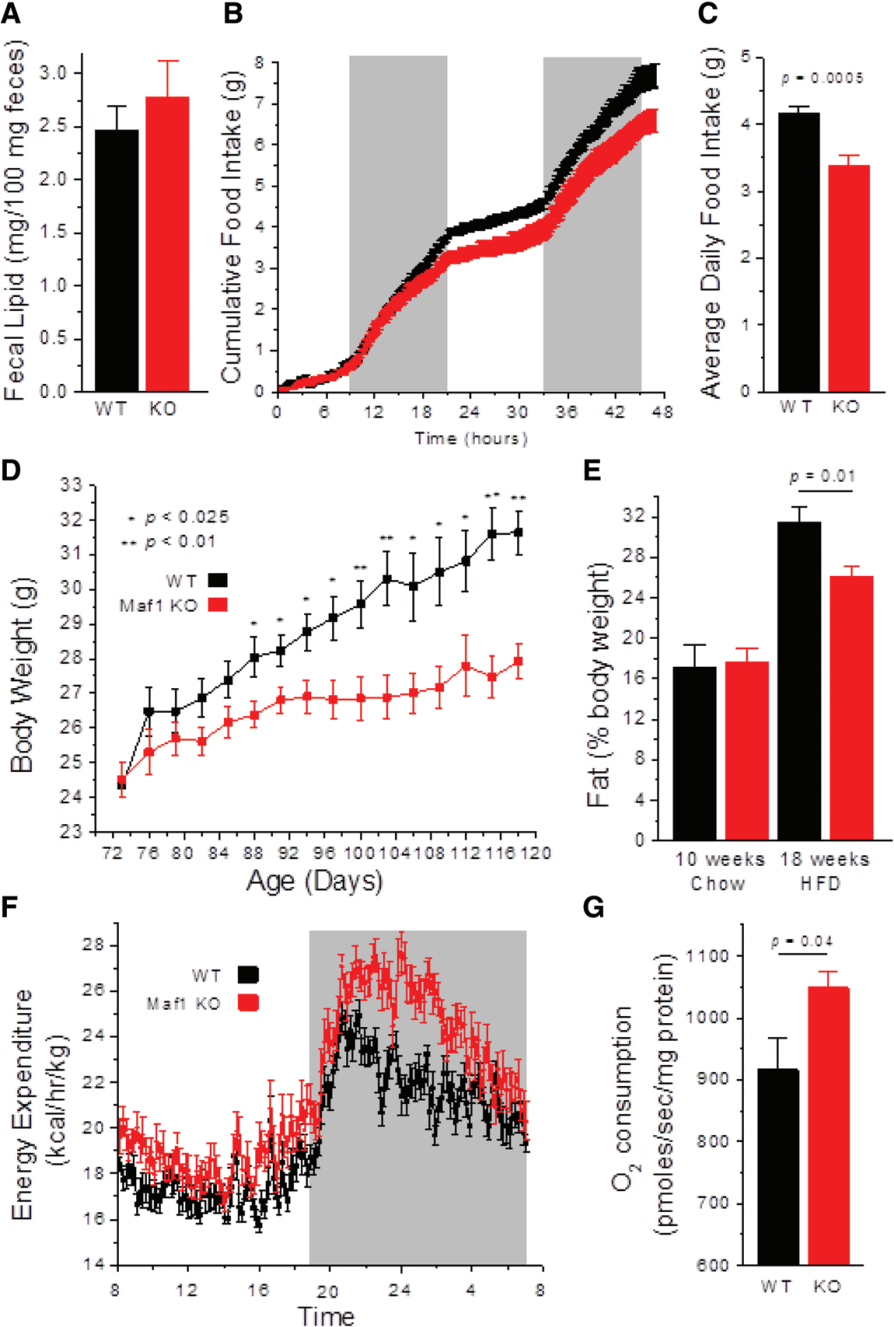
Hypophagia and metabolic inefficiency of *Maf1*^−/−^ mice. (*A*) Fecal lipid content of chow-fed mice (*n* = 4 per group). (*B*) Two-day cumulative food intake of weight-matched chow-fed mice (*n* = 4 per group). (*C*) Daily food intake of weight-matched mice averaged over 5 d. (*D*) Body weight curves of pair-fed mice on a HFD (*n* = 5 per group). (*E*) Percent body fat before and after 8 wk of HFD pair feeding (*n* = 5 per group). (*F*) Energy expenditure in HFD pair-fed animals (24-h averages over 5 d in metabolic cages; *n* = 4 per group; mice were 18 wk of age). (*G*) Oxygen consumption from mitochondrial complex 2 was measured on liver homogenates from HFD-fed mice (*n* = 8 per group). (Black) Wild-type (WT); (red) *Maf1*^−/−^ (KO). All values are presented as the mean ± SEM.

To explore possible factors contributing to the reduced weight of *Maf1*^−/−^ mice, we measured the fecal lipid content of chow-fed animals and found that malabsorption of dietary fat was not a significant factor in the lean phenotype of the knockout (Fig. 2A). We then performed feeding studies on weight-matched wild-type and *Maf1*^−/−^ animals and observed a reduction in food intake in the knockout mice (Fig. 2B,C). To determine whether this difference in behavior could explain the reduced body weight phenotype, we pair-fed animals on a HFD starting at 10 wk of age, a point at which there was no difference in the weight of the mice. Body weight curves diverged over 8 wk of HFD paired feeding, with *Maf1*^−/−^ mice being significantly lighter than wild-type mice under these conditions (Fig. 2D). The body fat content of *Maf1*^−/−^ mice increased following the switch to a HFD but remained significantly lower than for wild-type mice (Fig. 2E). Thus, while reduced food intake undoubtedly contributes to the lower weight of *Maf1*^−/−^ mice, it does not account entirely for this phenotype or the difference in body fat content. These observations suggested that wild-type and *Maf1*^−/−^ mice might show differences in energy expenditure. Indirect calorimetry studies of HFD pair-fed mice revealed an increase in the energy expenditure of *Maf1*^−/−^ animals, normalized for lean body mass, during both the day and night, with the differences being greater at night during the active period (Fig. 2F). The increase in energy expenditure was not the result of *Maf1*^−/−^ mice being more active, as locomotor activity determined by the number of infrared beam breaks in the metabolic chambers did not reveal any differences (Supplemental Fig. S3A). The respiratory exchange ratio was slightly elevated in *Maf1*^−/−^ mice during both the day and night, suggestive of a marginally enhanced use of glucose as an energy source (Supplemental Fig. S3B). Consistent with the increase in energy expenditure, liver homogenates of high-fat-fed *Maf1*^−/−^ mice showed increased O_2_ consumption by mitochrondrial complex 2 compared with wild-type controls (Fig. 2G). This occurred without any increase in mitochondrial DNA or changes in the cellular levels of selected proteins in the electron transport chain (Supplemental Fig. S3C,D). Thus, *Maf1*^−/−^ mice are resistant to obesity as a result of both reduced caloric intake (feeding) and increased energy expenditure. Notably, the diminished ability of *Maf1*^−/−^ mice to transform calories into biomass under pair feeding conditions (Fig. 2D) indicates that the animals are metabolically inefficient.

### Altered lipid homeostasis in livers and eWAT

**Figure 3.**
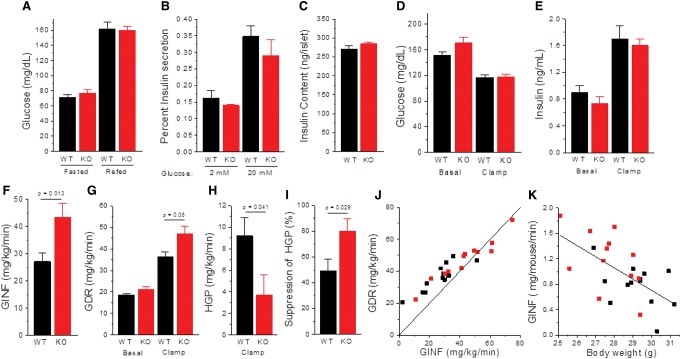
Blood glucose, insulin secretion, and analysis of insulin sensitivity. (*A*) Blood glucose concentrations were determined from tail vein bleeds after an overnight fast and following a 4-h refeed. Mice were 4 mo of age and were maintained on a breeder chow diet. (*n* = 7 mice per group). (*B*) Insulin secretion ex vivo was assayed in the presence of 2 mM and 20 mM glucose (five islets per well; *n* = 8 per condition per genotype; chow diet). Results are expressed as a percentage of the total insulin content of the islets used in the assay. (*C*) Insulin content of islets was calculated from five islets per sample (*n* = 16 per group). (*D*–*K*) Hyperinsulinemic–euglycemic clamp analysis of insulin sensitivity in 5-h-fasted mice. (*D*) Plasma glucose levels before and during the clamp. (*E*) Plasma insulin levels before and during the clamp. (*F*) Glucose infusion (GINF) rate needed to maintain euglycemia. (*G*) Glucose disposal rate (GDR) before and during the clamp was measured by the tracer dilution technique using [3-^3^H]glucose as tracer. (*H*) Hepatic glucose production (HGP) during the clamp. (*I*) Suppression of hepatic glucose production was calculated as the difference in HGP in the basal state (=basal GDR) and during the clamp divided by the basal HGP. (*J*) Rates of glucose disposal versus glucose infusion are shown for all of the mice in the study relative to a line with a slope of 1. HGP is the vertical difference between each data point and the line. (*K*) Glucose infusion rate (insulin sensitivity) is inversely correlated with the body weight of the mice. A linear fit is shown to all of the data. All values are presented as the mean ± SEM. Clamp data in *D*–*K* were obtained from 13 wild-type and 12 *Maf1*^−/−^ mice. (Black) Wild type (WT); (red) *Maf1*^−/−^ (KO).

Changes in metabolic efficiency could impact many processes, including glucose or lipid homeostasis. However, plasma glucose levels in the chow-fed state and after an overnight fast showed no differences between wild-type and *Maf1*^−/−^ mice at 4 mo of age, and pancreatic insulin content and islet insulin secretion ex vivo were normal (Fig. 3A–C). In a hyperinsulinemic–euglycemic clamp study, *Maf1*^−/−^ mice on a chow diet required higher glucose infusion rates to achieve euglycemia (Fig. 3D–F) and showed increased whole body glucose disposal as well as increased suppression of hepatic glucose production (Fig. 3G–J). These results indicate that *Maf1*^−/−^ mice are slightly more sensitive to insulin than control animals. However, these differences are associated with the lower body weight of *Maf1*^−/−^ mice (Fig. 3K). We conclude that insulin sensitivity is slightly increased in *Maf1*^−/−^ mice due to their lean phenotype rather than as a direct consequence of the absence of MAF1.

**Figure 4.**
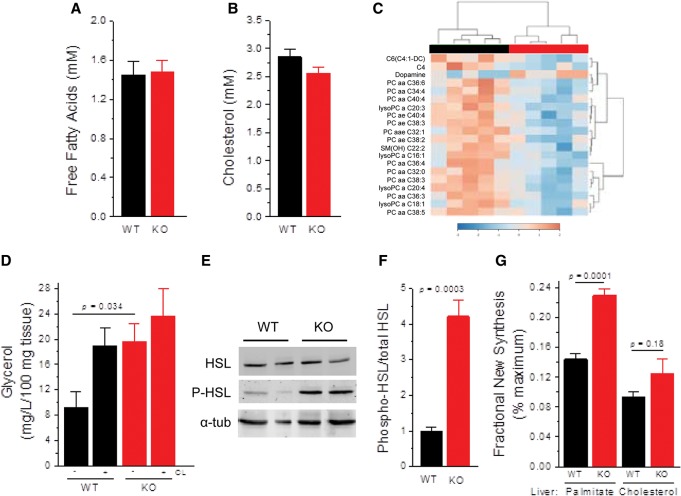
Altered lipid metabolism in *Maf1*^−/−^ mice. (*A*,*B*) Serum-free fatty acids and serum cholesterol were measured in overnight-fasted mice maintained on a standard chow diet (*n* = 6 per group). (*C*) Hierarchical clustering of plasma metabolite profiles from Biocrates AbsoluteIDQ p180 analysis performed with mice on a breeder chow diet. The top 20 metabolites by *t*-test (*P* < 0.025) were clustered using Pearson's correlation to measure similarity and Ward's linkage to minimize the sum of the squares of the clusters (MetaboAnalyst) (see also Supplemental Figure S4 and Supplemental Table S1). (*D*) Lipolysis in eWAT explants from mice on a breeder chow diet. Glycerol release in explants was measured in the presence and absence of the β3-adrenergic receptor agonist CL-316,243 (CL, *n* = 8 for wild-type under both treatments; *n* = 7 for untreated *Maf1*^−/−^; *n* = 4 for CL-316,243-treated *Maf1*^−/−^ explants). (*E*) Western blot of phospho-HSL (P-HSL) and total HSL from eWAT. (*F*) Quantitation of activated phospho-HSL over total HSL in eWAT (wild type, *n* = 8; *Maf1*^−/−^*, n* = 7; breeder chow diet). (*G*) De novo lipogenesis and cholesterol synthesis in livers from mice on a breeder chow diet were measured by tracer enrichment after 5 d of receiving 6% D_2_O in drinking water (*n* = 5 per group). All values are presented as the mean ± SEM.

Given the marked reduction in body fat in *Maf1*^−/−^ mice (Fig. 1B), we assayed for lipid metabolites in plasma. The concentrations of free fatty acids and cholesterol were normal in chow-fed knockout mice compared with controls (Fig. 4A,B). However, targeted metabolomics identified distinct differences between the two groups, including reductions in the levels of multiple glycerophospholipids in *Maf1*^−/−^ plasma (Fig. 4C; Supplemental Fig. S4; Supplemental Table S1). Basal lipolysis in eWAT explants was elevated in the knockout, comparable with wild-type tissue in which lipolysis was activated by the β3-adrenergic agonist CL-316243 (Fig. 4D). Notably, incubation of *Maf1*^−/−^ eWAT with the agonist did not substantially increase glycerol output over the untreated tissue, suggesting that lipolysis was close to maximally stimulated (Fig. 4D). Consistent with this possibility, the level of activated hormone-sensitive lipase (HSL) was greater than fourfold higher in eWAT of ad libitum chow-fed *Maf1*^−/−^ mice relative to controls (Fig. 4E,F). The observation that dopamine levels were elevated in the plasma of *Maf1*^−/−^ mice (Supplementary Table S1; Supplemental Fig. 4B) raises the possibility that activation of HSL may be achieved via Gαs-coupled dopamine D1 receptors on the adipocytes (Borcherding et al. 2011). Lipid metabolism was also altered in the livers of *Maf1*^−/−^ mice, which displayed increased de novo lipogenesis (Fig. 4G). Thus, *Maf1*^−/−^ mice exhibit altered lipid metabolism in the liver and WAT and a different profile of glycerophospholipids in plasma.

### Energy expenditure associated with futile synthesis of tRNA

To investigate global changes in gene expression in a tissue exhibiting *Maf1*^−/−^ phenotypes, deep sequencing was performed on eWAT RNA. Although MAF1 has been reported to regulate transcription of several protein-coding genes (Johnson et al. 2007; Palian et al. 2014), we did not identify any significant, reproducible changes in the Pol II transcriptome in this tissue (Supplemental Fig. S5A; Supplemental Table S2). Importantly, although the observed increase in energy expenditure could in principle reflect higher levels of adaptive thermogenesis, we observed no induction of known activators or markers of BAT or beige adipose tissue, including UCP1, which regulates proton leak in thermogenic tissues (Walden et al. 2012; Wu et al. 2012; Harms and Seale 2013). Indeed, Western blotting showed that UCP1 was unchanged in BAT and undetectable in eWAT (Supplemental Fig. S5B–D), and the body temperature and cold stress resistance of *Maf1*^−/−^ mice was normal (Supplemental Fig. S5E,F). These observations suggest that enhanced energy dissipation in *Maf1*^−/−^ mice does not result from recruitment of brown-like adipocytes in WAT or UCP1-mediated uncoupling of oxidative phosphorylation.

Previous studies of *Maf1* knockdown and overexpression in glioblastoma cells found inverse changes in the expression of the TATA-box-binding protein (TBP) at the RNA and the protein level that correlated with changes in RNA Pol I transcription (Johnson et al. 2007). MAF1 effects on TBP expression were due to its recruitment to the TBP promoter, whereas changes at the rDNA were thought to be mediated by the promoter selectivity factor SL1, which contains TBP as a central component (Grummt 2013). Although TBP expression was not affected in *Maf1*^−/−^ eWAT RNA (Supplemental Table S2), we examined this issue further in *Maf1*^−/−^ livers and also looked for changes in rDNA transcription (Supplemental Fig. S5G–I; Oie et al. 2014). No significant differences were detected in these assays. We infer that the effects of MAF1 on TBP expression and potentially other protein coding genes may be context-dependent.

**Figure 5.**
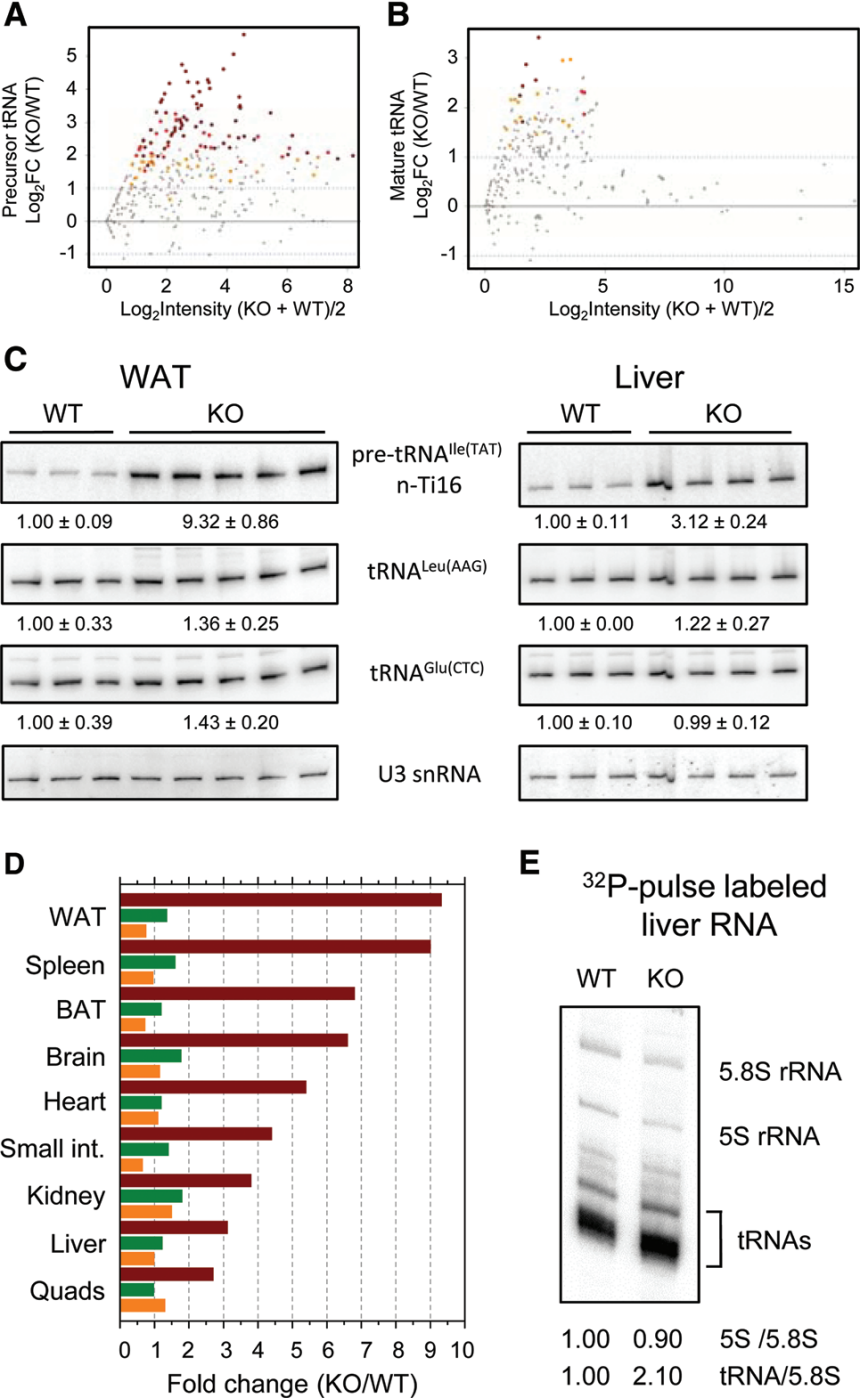
Futile cycling of tRNAs as a mechanism for energy expenditure. (*A*) Log ratio versus abundance (MA plot) of uniquely mapped precursor tRNA-specific RNA-seq reads in eWAT of breeder chow-fed mice (*n* = 3 per group). Yellow and red dots correspond to loci exhibiting significant changes called by limma or GLM, respectively. Brown dots correspond to loci with significant changes called by both methods. Gray dots correspond to loci with scores that are not statistically different. (*B*) MA plot of uniquely mapped mature tRNA reads in eWAT. The color scheme is the same as in *A*. (*C*) Northern blots of precursor and mature tRNA species from the eWAT and livers of breeder chow-fed mice. The fold change normalized to U3 snRNA is shown *below* each panel. (*D*) Precursor tRNA^Ile^ (TAT) n-Ti16 (maroon), mature tRNA^Leu^ (AAG) (green), and mature tRNA_i_^Met^ (CAT) (orange) levels were surveyed by Northern analysis in the indicated tissues of breeder chow-fed mice. The fold change was normalized to U3 snRNA. (*E*) Newly synthesized 5.8 S rRNA, 5S rRNA, and tRNAs from breeder chow-fed mice were quantified in total liver RNA following i.p. injection of ^32^P-orthophosphate and labeling for 4 h.

eWAT RNA sequencing (RNA-seq) data were analyzed to determine the effect of the knockout on the synthesis of precursor tRNAs. These molecules are short-lived and are widely used to assess the level of transcription by RNA Pol III (Upadhya et al. 2002; Michels et al. 2010). Consistent with the increase in polymerase occupancy of Pol III genes in *Maf1*^−/−^ tissue (Bonhoure et al. 2014), pre-tRNA-specific reads representing >100 different tRNA genes were markedly increased in the knockout (Fig. 5A; Supplemental Table S3). In contrast, mature tRNA-specific reads increased significantly for only a few tRNA genes, and the magnitude of these changes was much lower (Fig. 5B; Supplemental Table S3). Similar findings were obtained by Northern blotting of tRNA species from WAT and numerous other tissues (Fig. 5C,D; Table 1). For example, pre-tRNA^Ile^ (TAT) levels increased from approximately threefold in the liver and quadriceps to approximately ninefold in WAT and the spleen, while the levels of five different mature tRNAs, including initiator methionine tRNA (tRNA_i_^Met^), in various tissues showed only minor variations (Fig. 5C,D; Table 1). Additionally, quantitation of the tRNA fraction in the liver indicated only a modest 15% ± 3% increase in the knockout (*P* = 0.008, *n* = 7 per group), and total tRNA levels in a range of other tissues showed minimal changes (Supplemental Fig. 5J). Mature tRNA is reported to have a long (2- to 3-d) half-life in chicken livers and mouse uteruses (Miller 1973; Nwagwu and Nana 1980), so a large (greater than threefold) increase in Pol III transcription in the absence of MAF1 (Fig. 5A,C,D) should have been readily apparent in the steady-state abundance of the mature tRNA population. Since most mature tRNAs as well as bulk tRNA levels were not substantially affected (Table 1; Fig. 5B–D; Supplemental Fig. 5J), we conclude that increased tRNA synthesis in the knockout must be largely matched by increased turnover of nascent tRNA transcripts, pre-tRNAs, and/or mature tRNAs. Support for this view is provided by in vivo ^32^P pulse-labeling of liver RNA. Compared with the Pol I-derived 5.8S rRNA, labeling of Pol III-derived 5S rRNA was unaffected, and labeling of the newly synthesized mature tRNA population increased twofold (Fig. 5E); i.e., less than the threefold level measured for specific precursor tRNAs in this tissue (Table 1), suggesting that some turnover has occurred. In addition, since tRNA synthesis during the pulse was increased twofold but steady-state tRNA levels were not substantially changed (Supplemental Fig. 5J), tRNA turnover is again indicated. We conclude that increased synthesis and turnover of Pol III transcripts—most notably tRNAs, which account for ∼10% of total RNA—constitutes a futile cycle that is likely to be an important driver of energy expenditure in mice.

**Table 1.**
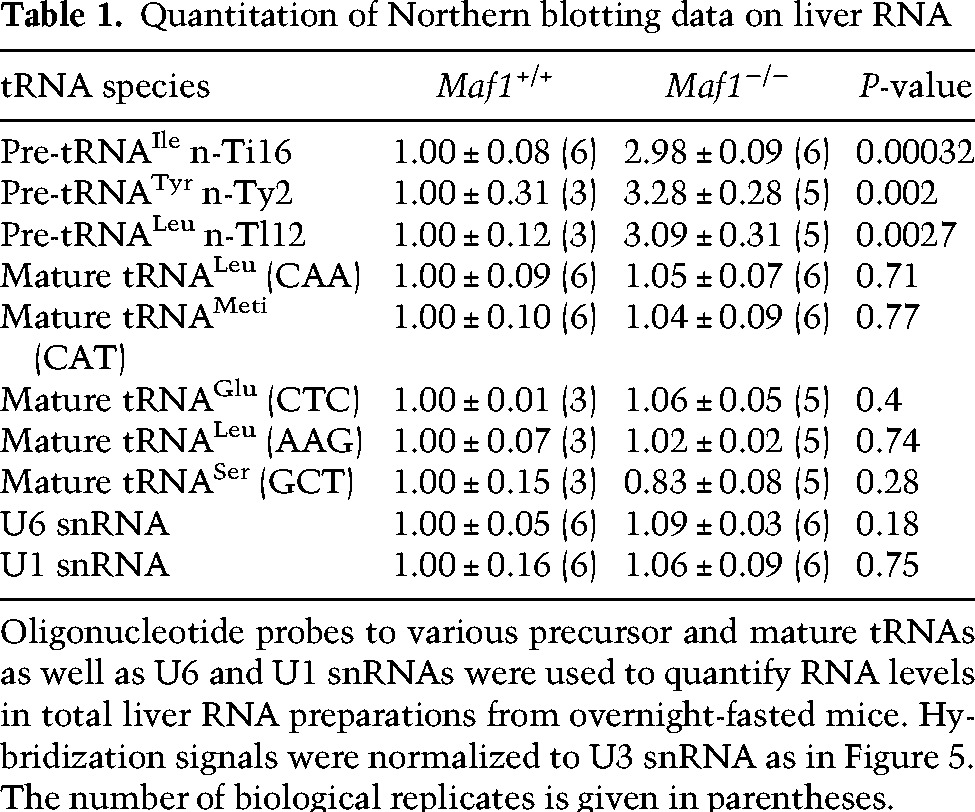
Quantitation of Northern blotting data on liver RNA

### Spermidine, autophagy, and life span extension

**Figure 6.**
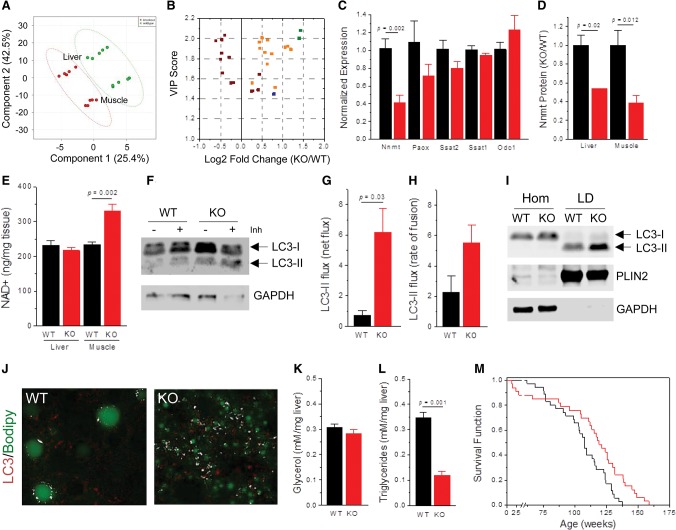
Elevated amino acid and polyamine levels in *Maf1*^−/−^ tissues correlate with induction of autophagy and life span extension. (*A*) Two-dimensional score plot of principal components from PLS-DA of liver and skeletal muscle metabolite profiles from mice on a breeder chow diet. The variance explained by each component is in brackets. Ellipses define regions of 95% confidence. (Green) Wild type (WT); (red) knockout (KO). (*B*) VIP scores (>1.0 is considered significant) obtained by PLS-DA are plotted against the fold change in metabolite concentration (normalized per milligram of tissue) in quadriceps. All metabolites had *P*-values <0.05 (*n* = 5 per group). (Green) Putrescine and spermidine; (orange) amino acids; (maroon) glycerolphospholipids; (blue) C5 acylcarnitine. (*C*) RT-qPCR analysis of polyamine pathway gene expression in the livers (*n* = 5 per group) of chow-fed mice. (*D*) Normalized NNMT protein levels from Supplemental Figure 6, C and D (*n* = 3 per group, chow-fed mice). (*E*) NAD^+^ levels in the liver and quadriceps as determined by LC-MS (*n* = 5 per group, breeder chow-fed mice). (*F*) Examination of autophagic flux in liver explants from mice on a breeder chow diet. The level of LC3-II, the lipidated autophagosome-associated form of LC3, was monitored in the presence or absence of lysosomal inhibitors (Inh). A representative blot is shown from three biological replicates per genotype. (*G*) Net flux shows the normalized difference in LC3-II ± Inh for each genotype (*n* = 3 per group). (*H*) Rate of autophagolysosome fusion compares the normalized ratio of LC3-II ± Inh. (*I*) Immunoblots of liver homogenates (Hom) and hepatic LD fractions. PLIN2 shows the equivalence of LD content, and GAPDH shows the lack of cytosolic contamination. (*J*) Colocalization (white) of BODIPY 493/503-stained LDs (green) and LC3 (red) in overnight-fasted livers. Images are at the same magnification and are representative of data from four wild-type and three *Maf1*^−/−^ mice. (*K*) Quantitation of liver glycerol (*n* = 4 per group). (*L*) Quantitation of liver triglycerides (*n* = 4 per group). The data in *F*–*L* were from the same cohort of breeder chow-fed mice. (*M*) Kaplan-Meier survival curves of female mice on a breeder chow diet (mean life span of wild type 113 wk, *n* = 35 [black]; mean life span of *Maf1*^−/−^ 121 wk, *n* = 33 [red]; *P* = 0.0054, log rank test; maximal life span assessed on the oldest quartile: 130 wk for wild type and 146 wk for *Maf1*^−/−^; *P* = 0.00013, *t*-test).

We hypothesized that a pervasive whole-body effect of the *Maf1* knockout on Pol III transcription and energy expenditure was likely to generate a common metabolic signature in different tissues. To assess this possibility, we conducted targeted metabolite profiling in liver and skeletal muscle. Multivariate partial least squares discriminant analysis (PLS-DA) of the aggregated data showed that wild-type and knockout tissues are readily distinguished by their metabolite profiles (Fig. 6A). Multiple statistical measures (variable importance in projection [VIP] scores and *t*-tests) indicate that *Maf1*^−/−^ tissues have significantly elevated levels of many amino acids and polyamine pathway metabolites, including ornithine, putrescine, and spermidine (Fig. 6B; Supplemental Fig. S6B; Supplemental Table S1). Perturbations of polyamine synthesis have been linked to changes in adiposity (Jell et al. 2007; Pirinen et al. 2007), and mice expressing reduced levels of nicotinamide *N*-methyltransferase (NNMT), which influences polyamine synthesis, are obesity-resistant (Kraus et al. 2014). Consistent with these observations and the increased level of spermidine in *Maf1*^−/−^ tissues, the expression of *Nnmt* mRNA was significantly reduced in the liver, as was the level of NNMT protein in the liver and muscle (Fig. 6C,D; Supplemental Fig. S6C,D). NNMT methylates nicotinamide using S-adenosyl methionine (SAM) as a methyl donor. Thus, in addition to its potential to affect SAM-dependent methylation reactions and the supply of propylamine groups for polyamine synthesis, NNMT can regulate the availability of NAD^+^ for cellular redox metabolism (Supplemental Fig. S6A; Kraus et al. 2014). To examine this issue, we measured the total cellular concentration of NAD^+^ in wild-type and *Maf1*^−/−^ livers and skeletal muscle by liquid chromatography-mass spectrometry (LC-MS). Consistent with the view that NAD^+^ synthesis in the liver is not limited by the activity of the nicotinamide salvage pathway (Houtkooper et al. 2010; Kraus et al. 2014), the level of NAD^+^ in *Maf1*^−/−^ livers was the same as for wild-type tissue (Fig. 6E). In contrast, the level of NAD^+^ was increased ∼40% in *Maf1*^−/−^ muscle (Fig. 6E). Similar increases have been reported in the muscles of mice with a whole-body deletion of poly(ADP-ribose) polymerase, a major consumer of NAD^+^, and in mice whose diet has been supplemented with the NAD^+^ precursor nicotinamide ribonucleoside (Cantó et al. 2012; Houtkooper et al. 2012). Importantly, these animal models have increased energy expenditure and are protected from HFD-induced obesity. In light of these studies, it appears likely that altered NAD^+^ metabolism contributes to the obesity resistance of *Maf1*^−/−^ mice.

Spermidine is a known inducer of autophagy in yeast, flies, worms, and mammalian cells, and enhanced autophagy is critical for the life span-extending effects of spermidine in these organisms (Eisenberg et al. 2009). Accordingly, livers from overnight-fasted *Maf1*^−/−^ mice displayed increased autophagic activity compared with controls, as shown by the elevated delivery of LC3-II-positive autophagosomes to lysosomes (net LC3-II flux) and their subsequent lysosomal fusion (Fig. 6F–H; Klionsky et al. 2007). Lysosomal degradation of proteins is consistent with the observed increase in amino acid levels (Fig. 6B; Supplemental Fig. S6B; Supplemental Table S1). In addition, enhanced autophagic activity in the knockout was also associated with increased colocalization of LC3 with BODIPY-stained LDs and increased sequestration of hepatic LDs by LC3-II-positive autophagosomes (Fig. 6I,J). Moreover, we observed a significant reduction in hepatic triglycerides but not free hepatic glycerol in *Maf1*^−/−^ mice (Fig. 6K,L). Together, these findings indicate activated mobilization of hepatocellular lipids via lipophagy (Singh et al. 2009). Increased lipid consumption through autophagy together with the increase in de novo lipogenesis in the *Maf1*^−/−^ liver (Fig. 4G) reveals contributions to metabolic inefficiency and energy expenditure in the mice via increased futile cycling of hepatic lipids. Finally, Kaplan-Meier survival curves revealed a statistically significant extension of mean and maximal life span for female *Maf1*^−/−^ mice, in accordance with the effects of spermidine and autophagy in other model organisms (Fig. 6M; Eisenberg et al. 2009). A similar trend was observed for male mice (Supplemental Fig. S6E).

## Discussion

Our work shows that the loss of *Maf1*, a ubiquitous global regulator of transcription by RNA Pol III, significantly impacts whole-body metabolism and energy expenditure while providing health benefits. These benefits include resistance to diet-induced obesity and nonalcoholic fatty liver disease as well as an extension of life span that can be attributed to previously documented effects of spermidine on the induction of autophagy and longevity in yeast, flies, and worms (Eisenberg et al. 2009). Obesity resistance in *Maf1*^−/−^ mice is achieved through reduced food intake and increased metabolic inefficiency. In this study, we focused on the metabolic component. At the whole-body level, *Maf1*^−/−^ mice demonstrate metabolic inefficiency by their reduced growth rate under paired-feeding conditions (i.e., lower body weight despite equal caloric intake). At the molecular level, metabolic inefficiency is apparent from the increased synthesis and turnover (futile cycling) of tRNAs and hepatic lipids. We infer that the increased energetic cost of these processes alters the balance between fuel utilization and storage and contributes to the lean phenotype. Additional factors contributing to the energy expenditure and obesity resistance of the mice include the reduced expression of NNMT in the liver, muscle, and potentially other tissues and downstream effects on NAD^+^ metabolism and/or the polyamine pathway (Kraus et al. 2014; Liu et al. 2014). How the different molecular effects of the *Maf1* knockout are partitioned in terms of energy expenditure and obesity resistance has yet to be determined, but our current results already establish MAF1 as a novel and unconventional therapeutic target for the treatment of obesity and related diseases.

Reduced expression of *Nnmt*, which we observed in the liver and muscle but not in eWAT, can increase the level of SAM and/or SAM/SAH ratios and the supply of nicotinamide (Supplemental Fig. S6A; Kraus et al. 2014). Increased SAM levels in turn can lead to increased polyamine synthesis, and, indeed, the liver and muscle showed increased levels of polyamine pathway metabolites, including spermidine. Perturbations of polyamine pathway flux are associated with obesity resistance or sensitivity in several mouse models (Jell et al. 2007; Pirinen et al. 2007; Kraus et al. 2014; Liu et al. 2014). In addition to oligonucleotide-directed knockdown of *Nnmt*, which confers obesity resistance primarily by affecting *Nnmt* expression in the liver and adipose tissue (Kraus et al. 2014), whole-body changes in polyamine catabolism have been engineered by overexpression and underexpression of spermidine/spermine-*N*^*1*^-acetyltransferase (SSAT) (Supplemental Fig. S6A). By increasing or decreasing polyamine acetylation in these models, acetyl-CoA and malonyl CoA pools were altered with corresponding changes in fatty acid synthesis, fatty acid oxidation, and body fat accumulation. These studies implicate enhanced polyamine pathway cycling in the obesity resistance of *Maf1*^−/−^ mice.

Elevated nicotinamide supply resulting from decreased *Nnmt* can lead to increased NAD^+^ levels (Kraus et al. 2014), which we observed in muscle tissue. Multiple genetic and pharmacological interventions that raise the level of NAD^+^ are known to enhance oxidative metabolism and provide protection against diet-induced obesity (Bai et al. 2011; Cantó et al. 2012; Pirinen et al. 2014). Along with its role as a cofactor in oxidoreductase reactions, NAD^+^ is a rate-limiting cosubstrate for the sirtuin family of NAD^+^-dependent deacetylases, which regulate the activity of several key transcription factors controlling nuclear and mitochondrial metabolism (Houtkooper et al. 2012). Our RNA-seq analysis of *Maf1*^−/−^ eWAT did not find any significant changes in gene expression for SIRT1 target genes (or other protein-coding genes), but since *Nnmt* expression was not affected in this tissue, NAD^+^ levels may not have been elevated. Gene expression and metabolic profiling of additional *Maf1*^−/−^ tissues will further clarify the relationship between NNMT, NAD^+^, and metabolism in these mice.

The finding that the synthesis of precursor tRNAs can be increased 10-fold or more, depending on the gene and the tissue, without significant changes at the level of mature tRNA suggests the existence of a robust homeostatic mechanism to prevent the global accumulation of these molecules. Although we did not explore the mechanism of tRNA turnover, it seems likely that the large amount of precursor tRNAs generated in the *Maf1* knockout leads to defects in tRNA processing and/or modification, and such molecules—in particular hypomodified tRNAs—are known to be rapidly degraded (Kadaba et al. 2004; Alexandrov et al. 2006; Chernyakov et al. 2008; Wilusz et al. 2011). Even with rapid tRNA turnover largely offsetting elevated precursor tRNA synthesis, subtle changes in the composition of the tRNA pool in the knockout are likely (Dittmar et al. 2006; Ciesla et al. 2007; Pang et al. 2014), and this in turn has the potential to alter the expression of genes whose codon usage is sensitive to these changes (Gingold et al. 2014). The recent identification of unique translational programs for proliferation and differentiation genes that reflect differences in codon usage and corresponding changes in the tRNA pool (Gingold et al. 2014) may well apply in other situations such as metabolic disease and the response to stress. In this regard, it is likely significant that the effect of deleting *Maf1* on Pol III gene transcription is not equal among different tissues and thus may lead to tissue-specific effects on gene expression.

The lower growth rate of *Maf1*^−/−^ mice is consistent with their reduced feeding and metabolic inefficiency but is strikingly different from the increased cell growth and accelerated larval development seen upon *Maf1* knockdown in *Drosophila* (Rideout et al. 2012). Importantly, these *Drosophila* phenotypes were recapitulated in flies overexpressing tRNA_i_^Met^, which promoted growth by stimulating protein synthesis. Our examination of tRNA_i_^Met^ levels in multiple mouse tissues found no evidence of its overexpression and thus provides a logical explanation for the growth-related phenotypic difference.

The normal levels of tRNA_i_^Met^ in *Maf1*^−/−^ mice are also consistent with these mice not being prone to tumorigenesis, as demonstrated by their extended life span. Until now, the tumorigenic potential of a *Maf1* knockout in mammals has been an open, intriguing question given (1) substantial correlative data linking deregulated Pol III transcription and increased tRNA_i_^Met^ levels to cell transformation and tumorigenesis (White 2008; Pavon-Eternod et al. 2013), (2) the requirement for elevated levels of Pol III transcripts for Myc-driven tumorigenesis (Johnson et al. 2008), and (3) the ability of *Maf1* overexpression to suppress anchorage-independent growth of transformed cells and tumor formation in a xenograft mouse model (Johnson et al. 2007; Palian et al. 2014). While the mouse *Maf1* knockout does not promote tumorigenesis or add to the role of Pol III transcription in the development of cancer, the likely explanation and important insight is that not all interventions that increase Pol III synthesis are capable of increasing the level of tRNA_i_^Met^ or other mature tRNAs.

Studies in mammalian cells and worms have recently reported that perturbing the expression of MAF1 affects lipogenesis (Khanna et al. 2014; Palian et al. 2014). Notably, RNAi-mediated knockdown of MAF1 increased de novo lipogenesis in these studies, consistent with our observations in the livers of *Maf1*^−/−^ mice (Fig. 4G). However, the apparent mechanism and the net effect on lipid accumulation in these studies differ from our findings. Whereas Maf1 overexpression or knockdown had reciprocal effects on the mRNA levels of key lipogenic enzymes (e.g., fatty acid synthase [FASN]) in worms and mammalian cells (Khanna et al. 2014; Palian et al. 2014), we did not detect significant changes in FASN expression in the liver by either RT-qPCR or Western blotting (RD Moir and A Byrnes, unpubl.). Also, liver triglycerides were lower (Fig. 6L), not higher, in the knockout mice, in keeping with the induction of lipophagy, the increase in whole-body energy expenditure, and the lean phenotype. At the present time, we can only speculate about the basis of the differences between our results and these other studies. Possibilities include partial versus complete ablation of *Maf1*, differences in diet and/or nutrients supplied in growth media, and effects due to short-term versus long-term changes in gene expression and/or metabolism.

The obesity and fatty liver disease resistance of the whole-body *Maf1* knockout may have, in part, a basis in metabolic inefficiency similar to that of the liver-specific knockout of NML. Hepatic NML deficiency leads to obesity resistance due to a failure to repress rDNA transcription by RNA Pol I, the main consumer of nucleotides among the RNA polymerases (Oie et al. 2014). This effect is extreme on a HFD. Thus, it appears that the normal function of NML and MAF1 in transcriptional repression by RNA Pol I and Pol III, respectively, is critical for the conservation of metabolic energy and the storage of excess calories as fat. The liver-specific phenotype of the NML knockout coupled with differences in nucleotide consumption between Pol I and Pol III (∼60% vs. 15% in growing cell populations) argues that a knockout of *Maf1* in any single tissue is unlikely to generate the full complement of phenotypes or provide the same level of protection against diet-induced obesity as the whole-body knockout. For MAF1, the overall health benefit is likely to be derived from reducing food intake and spreading the increase in energy expenditure over virtually every cell in the body.

In summary, our findings indicate that obesity resistance in *Maf1*^−/−^ mice is achieved through multiple mechanisms. In addition to the loss of repression of RNA Pol III transcription and the futile cycling of tRNAs in the whole animal, other contributions to energy expenditure are provided by the futile cycling of hepatic lipids and potentially polyamines, with enhanced oxidative metabolism enabled by the elevated level of NAD^+^. Both direct and indirect effects of the *Maf1* knockout on gene expression are involved, and we suggest that changes in some processes may be driven by an increase in the demand for nucleotides. Finally, we note that the lower caloric intake of the mice may be due to the loss of MAF1 function in the brain and/or may reflect a differential response of the CNS to factors secreted from peripheral *Maf1*^−/−^ tissues.

## Materials and methods

### Animals

All experiments involving mice were performed using protocols approved by the Institutional Animal Care and Use Committee (IACUC) of the Albert Einstein College of Medicine or the Veterinary Office of the Canton of Vaud (SCA-EXPANIM, Service de la Consommation et des Affaires Vétérinaires, Epalinges, Switzerland) in accordance with the Federal Swiss Veterinary Office guidelines. *Maf1*^+/−^ mice were generated in the C57Bl/6J background (Ozgene). Details of the targeting vector, breeding, housing, and diets are given in the Supplemental Material. All experiments were performed with male mice except for body weight (growth rate) and life span studies, which were performed with animals of both sexes.

### Histology and cell size and cell number determination

Adipose and liver samples were fixed in 10% buffered formalin prior to paraffin-embedding, sectioning, and staining by hematoxylin and eosin (H&E). Livers were frozen in OCT cryo-embedding medium for Oil-Red-O staining. Adipose cell volume was determined from measurements of the cell radius (>250 cells per condition, two mice per genotype, *v* = 4/3π*r*^3^). Error estimates of the cell radius were propagated to volume as 4π*r*^2^. Cell numbers were determined by dissecting and weighing eWAT fat pads from chow-fed mice (12 mo of age). A tissue sample (100 mg) was digested with collagenase, the mixture was centrifuged at 200*g* for 10 min, and the cells in the supernatant were counted in a hemocytometer.

### Body composition, fecal lipids, and indirect calorimetry

Body composition was determined by EchoMRI. The lipid content of mouse feces was determined by gravimetry with [^14^C] triolein as a radioactive tracer to normalize for the recovery of neutral lipids (Argmann et al. 2006). Measurements of food intake, oxygen consumption, CO_2_ production, respiratory exchange ratio (RER), and locomotor activity were performed using an indirect calorimetry eight-cage system (Oxymax) as described in the Supplemental Material.

### Mitochondrial respiration

Oxygen consumption in liver homogenates of HFD-fed mice was measured using the Oxygraph-2k (Oroboros Instruments). Mitochondrial complexes 1 and 2 were stimulated by injection of 5 mM pyruvate, 2 mM malate, 10 mM glutamate, 2.5 mM ADP, and 10 mM succinate followed by inhibition of the mitochondrial complex 1 by injection of 0.5 μM rotenone.

### Insulin secretion and content

Pancreatic islets were isolated from 17-wk-old mice by digestion with collagenase and separation of exocrine tissue (Gotoh et al. 1987). After 24 h in suspension culture, the islets were distributed into wells (five islets per well) of a 12-well plate, incubated for 1 h at 37°C at a low-glucose concentration (2.8 mM), transferred into wells containing 2 or 20 mM glucose in triplicate, and incubated for another hour. The islets were then separated from the supernatant and lysed in acidic ethanol to extract the insulin. Insulin content of the islets and the supernatant was determined, and insulin secretion was expressed as percent of insulin content.

### Hyperinsulinemic–euglycemic clamp

A dual tracer clamp ([3-^3^H]glucose infusion and 2-deoxy-d-[1-^14^C] glucose bolus) was performed in 3-mo-old male mice. Mice received an indwelling silicone catheter in the femoral vein and were allowed to recover for 4–7 d before a hyperinsulinemic–euglycemic clamp study was conducted (see the Supplemental Material). Rates of basal and insulin-stimulated glucose disposal and hepatic glucose production were determined by the [3-^3^H]glucose dilution method.

### Metabolite profiling

Biocrates AbsoluteIDQ p180 analysis of metabolites in plasma was performed according to the manufacturer's instructions. Plasma was prepared from retroorbital bleeds of overnight-fasted mice (19 wk of age). Tissue samples from overnight-fasted mice (22–24 wk of age) were freeze-clamped in liquid nitrogen and ground to powder with a mortar and pestle on dry ice. The powdered tissue (50–100 mg) was extracted for analysis. NAD^+^ levels were determined by LC-MS. Metabolites with CVs >30% were excluded from the analysis. Supervised PLS-DA was performed using MetaboAnalyst or SIMCA-P software.

### Assays of lipogenesis and lipolysis

Lipogenesis and cholesterol synthesis were measured with deuterated water as a tracer (Vaitheesvaran et al. 2012). Mice were provided with 6% D_2_O in their drinking water for 5 d. Palmitate in liver triglyceride and cholesterol was analyzed by gas chromatography-MS (GC-MS) to determine D_2_O enrichment relative to body water. Lipolysis assays were performed on epididymal fat pads harvested from preprandial ad libitum-fed mice (Marcelin et al. 2012). Glycerol concentration was measured using a colorimetric assay kit (Cayman Chemical).

### Tissue extracts and Western blotting

BAT and WAT were homogenized in lysis buffer (50 mM Tris-HCl at pH 7.4, 1 mM EDTA, 1 mM EGTA, 50 mM NaF, 10 mM sodium glycerophosphate, 20 mM sodium pyrophosphate) containing Complete Mini and PhosSTOP (Roche). Samples were spun at 14,000*g* for 15 min at 4°C, and the interphase was transferred to a new tube. Triton was added to 1% (v/v), and samples were incubated for 30 min at 4°C with agitation and then centrifuged as above to obtain the supernatant. Extracts from other tissues were prepared in RIPA buffer with inhibitors. Protein concentrations were determined by BCA assay (Pierce). Details of the antibodies used are provided in the Supplemental Material.

### RNA-seq analysis

Epididymal adipose tissue was harvested and freeze-clamped in liquid nitrogen from overnight-fasted 22- to 24-wk-old mice maintained on a breeder chow diet. Total RNA was prepared (Qiagen miRNeasy) and digested with DNase I, and RNA quality was assessed by capillary electrophoresis (Agilent 2100 Bioanalyzer). Library preparation and directional RNA-seq were performed at the Einstein Epigenomics Core Facility. Data analysis is described in the Supplemental Material. RNA-seq data have been deposited in NCBI's Gene Expression Omnibus under accession number GSE65976.

### Northern blotting and in vivo labeling of RNA

Tissue samples (50–100 mg, flash-frozen in liquid N_2_) were homogenized into Qiazol lysis reagent (Qiagen), and RNA was purified according to the manufacturer's directions. RNA was precipitated twice, quantified, and resolved by denaturing polyacrylamide electrophoresis before electrophoretic transfer to Nytran Plus membranes (GE Healthcare) and hybridization with [^32^P]-end labeled oligonucleotide probes at 42°C (Li et al. 2000). tRNA signals detected by phosphorimaging were quantified and normalized to U3 snRNA to compare expression in wild-type and knockout samples. For in vivo labeling, mice (23 wk of age) maintained on a breeder chow diet were fasted overnight and injected i.p. with 0.5 mCi ^32^P-orthophosphate (carrier-free) in Tris-buffered saline. After 4 h, the animals were sacrificed, tissues were dissected and freeze-clamped, and total RNA was prepared for electrophoresis on denaturing polyacrylamide gels.

### Autophagy assays

In vivo LC3 autophagic flux analyses determined the amount of LC3-II that accumulates in lysosomes when exposed to lysosomal inhibitors, 20 mM ammonium chloride, and 100 μM leupeptin. Briefly, freshly harvested liver explants were rapidly placed in dishes containing high-glucose DMEM in the presence or absence of inhibitors and transferred to a CO_2_ incubator for 2 h at 37°C and 5% CO_2_. Following incubation, explant lysates were generated and subjected to immunoblotting for LC3. Autophagic flux, expressed as rate of autophagolysosome fusion, was determined by the ratio of normalized intensities for LC3-II in inhibitor-treated versus untreated explants. Net flux was determined by subtracting the normalized intensity of untreated LC3-II from the corresponding inhibitor-treated value. Mouse liver LD fractions were isolated as previously described (Singh et al. 2009). Methods for immunohistochemistry are described in the Supplemental Material.

### Liver glycerol and triglyceride analyses

Liver glycerol was measured in tissue aqueous homogenates. Triglyceride content was analyzed in liver samples subjected to lipid extraction in a 2:1 chloroform:methanol mixture containing 0.05% sulfuric acid for 24 h at −20°C. Tissue glycerol and triglyceride analyses were carried out using a commercial kit from Sigma-Aldrich as per the manufacturer's instructions.

### Statistics

Results are expressed as mean ± SEM. Differences between animals and/or treatments were tested for statistical significance using Student's unpaired *t*-test unless otherwise indicated.

## Supplementary Material

Supplemental Material
