# Supplementary material for: Loss of the RNA polymerase III repressor MAF1 confers obesity resistance
Source: Genes Dev. 2015 May 1;29(9):934–47. doi: 10.1101/gad.258350.115 (PMC4421982; doi:10.1101/gad.258350.115)
Supplement: Supplemental Material [file supp_29_9_934__index.html]

Supplemental Material 

# Loss of the RNA polymerase III repressor MAF1 confers obesity resistance

## Supplemental Material

**Files in this Data Supplement:**

- Supp Material.pdf
- Supp Table S1.xlsx
- Supp Table S2.xlsx
- Supp Table S3.xlsx
