# Supplementary material for: Loss of the RNA polymerase III repressor MAF1 confers obesity resistance
Source: Genes Dev. 2015 May 1;29(9):934–47. doi: 10.1101/gad.258350.115 (PMC4421982; doi:10.1101/gad.258350.115)

SUPPLEMENTAL MATERIAL LIST

Figure S1. Targeting, genotyping, breeding and phenotyping. Related to Figure 1.

Figure S2. Body weights of *Maf1* null mutant mice created using a targeted zinc finger nuclease. Related to Figure 1.

Figure S3. Locomotor activity, respiratory exchange ratios, mitochondrial copy number and complex abundance. Related to Figure 2.

Figure S4. Targeted metabolite profiling of plasma. Related to Figure 4.

Figure S5. *Maf1*$^{-/-}$ mice do not expend energy by inducing UCP1-mediated adaptive thermogenesis or activating rRNA synthesis. Related to Figure 5.

Figure S6. Polyamine pathway, liver metabolites and lifespan of male wild-type and *Maf1*$^{-/-}$ mice. Related to Figure 6.

Table S1. Metabolite concentrations in plasma, skeletal muscle and liver. Related to Figure 4 and Supplemental Figures S4 and S6.

Table S2. RNA-seq analysis of wild-type and Maf1 KO eWAT. Related to Supplemental Figure S5.

Table S3. Pol III transcriptome RNA-seq scores. Related to Figure 5.

SUPPLEMENTAL MATERIALS AND METHODS

SUPPLEMENTAL REFERENCES

SUPPLEMENTAL FIGURE LEGENDS

Figure S1. Targeting, genotyping, breeding and phenotyping. (A) Schematic of the construct used to direct homologous recombination at the *Maf1* locus in ES cells. (B) PCR genotyping of tail DNA. Diagnostic products for *Maf1*$^{+/+}$ (421 bp) and *Maf1*$^{-/-}$ (376 bp). (C) RT-qPCR of *Maf1* and β-actin mRNAs from wild type and *Maf1*$^{-/-}$ mouse embryo fibroblasts (Reina et al., 2006). n.d. : not detected. (D) Frequency of genotypes from heterozygous crosses. (E) Fertility and fecundity in homozygous crosses. (F) Body weights of *Maf1*$^{+/+}$ and *Maf1*$^{-/-}$ female mice fed *ad libitum* on breeder chow (mean ± s.e.m.). (G) H&E-stained sections of eWAT and oil red O-staining of liver are compared as indicated. Images are at the same magnification and include a 100 μm scale bar. Average cell diameters and estimated cell volumes were determined from multiple fields (see Materials and Methods and Fig. 1I).

Figure S2. Body weights of *Maf1* null mutant mice created using a targeted zinc finger nuclease. (A) Schematic of the *Maf1* locus before and after zinc finger nuclease genome editing. The targeted *Fok*1 cutting site in the wild type *Maf1* sequence is indicated in red. Edited alleles are indicated in blue and the new amino acid sequences are in green followed by a asterisk representing the stop codon. Green and red polygons correspond to the start and the end of the wild type *Maf1* coding sequence. (B) Body weights of male wild type and *Maf1* null mice from homozygous crosses fed *ad libitum* on a high fat diet (mean ± s.e.m.).

Figure S3. Locomotor activity, respiratory exchange ratios, mitochondrial copy number and complex abundance. (A) Total locomotor activity (IR beam breaks in the X and Z dimensions) of high fat pair-fed mice during the light and dark cycle (daily averages from 5 days

in metabolic cages, $n = 4$ per group). (B) Respiratory exchange ratios of high fat pair-fed mice during the light and dark cycle (daily averages from 3 days in metabolic cages, $n = 4$ per group) (C) Mitochondrial copy number in mouse liver was calculated using two different mitochondrial genes (CytB and ND1) relative to the nuclear H19 gene ($n = 9$ per group). (D) Representative proteins in different complexes of the electron transport chain were examined in total mouse liver homogenates using MitoProfile® Total OXPHOS Rodent WB Antibody Cocktail.

Figure S4. Targeted metabolite profiling of plasma. The Biocrates Absolute*IDQ* p180 system was used to quantify the levels of 186 metabolites representing glycerophospholipids, sphingolipids , acyl carnitines, amino acids and other biologic amines. Mice (4 months of age) were fasted overnight and retro-orbital bleeds were taken to prepare plasma. Multivariate partial least squares discriminant analysis (PLS-DA) was performed (MetabolAnalyst) on 159 metabolites that returned values for each of the five wild-type and five knockout plasma samples. (A) 2D score plot of principal components 1 and 2 shows that wild-type and *Maf1*$^{-/-}$ mice are readily distinguished by their plasma metabolite profiles (ellipses define the region of 95% confidence). Leave one out cross validation indicates that the data is best described by a single-component (accuracy 0.8, R2 0.88, Q2 0.54). (B) Fold change in plasma metabolites (KO/WT). The top 31 scoring plasma metabolites are rank ordered, top to bottom, by p value (p values < 0.05). Metabolite concentrations and p values are provided in Supplemental Table S1.

Figure S5. *Maf1*$^{-/-}$ mice do not expend energy by inducing UCP1-mediated adaptive thermogenesis. (A) RNA-seq analysis of eWAT. The MvA plot shows the log2 fold change (KO/WT) versus the log2 average abundance (counts per million reads) for uniquely mapped

reads obtained from biological triplicate RNA samples. Statistical analysis of differential gene expression was performed using EdgeR (Supplemental Table S2). The output was filtered by requiring a >two-fold difference in gene expression, an adjusted p value < 0.05 (EdgeR) and >50 reads (normalized) per KO sample for up-regulated genes or >50 reads per WT sample for down-regulated genes. The 13 genes satisfying these criteria are highlighted in red. Due to the sensitivity of EdgeR to outliers, differential gene expression was also evaluated using DESeq. None of the 13 genes scored as statistically significant using this approach. (B) Western blotting of UCP1 and β tubulin in brown adipose tissue. Animals were housed at 22°C and fed *ad libitum* on a breeder chow diet. (C) UCP1 signal intensities from panel B were quantified and normalized to β tubulin. (D) Western blotting of UCP1 in white adipose tissue. Animals were raised as in panel B. BAT lysate was loaded as a positive control. (E) Body temperature of animals housed at 22 ºC (n = 7 mice per group). (F) A cold stress test was performed by placing the mice at 4 ºC in individual cages without bedding or food and measuring their body temperature using a rectal thermocouple microprobe (Physiotemp IT-23) at hourly intervals. Four mice at 5 months of age were used per group. WT, blue; KO, orange) (G) Quantitation of TBP expression in total liver RNA was performed as in Fig. 6C (n = 5 per group). (H) Western blotting of TBP in nuclear extracts from WT and Maf1 KO liver. (I) Pre-rRNA levels in liver were calculated in the chow-fed and the fasted (16 hr) state by RT-qPCR using primers specific for the 5' external transcribed spacer with normalization to GAPDH (n = 3 WT fed, n = 2 WT fasted and KO fed, n = 4 KO fasted). Comparable results were obtained using cyclophilin for normalization. (J) Quantitation of total tRNA ratios (KO/WT) relative to 5.8S rRNA in different tissues from ethidium bromide-stained denaturing polyacrylamide gels (n = 7 per group for liver,

$n = 2$ per group for eWAT and brain, other tissues represent single determinations). Values are presented as the mean ± SEM.

Figure S6. Polyamine pathway, liver metabolites and lifespan of male wild-type and *Maf1*$^{-/-}$ mice. (A) Polyamine biosynthetic pathway showing inputs from amino acids, methionine, arginine and ornithine along with enzymes and cofactors (Ac-CoA, acetyl coenzyme A; SAM, S-adenosyl methionine, dcSAM, decarboxylated SAM; SAH, S-adenosyl homocysteine; Nam, nicotinamide; Me-Nam, N-methyl Nam). The ability of NNMT (green) to influence polyamine (blue) synthesis by affecting the availability of SAM is indicated. (B) Variable importance in projection (VIP) scores (>1.0 is considered significant) obtained by PLS-DA of liver metabolite profiles (Biocrates p180) are plotted against the fold change in metabolite concentration (normalized per mg tissue) ($p < 0.05$, $n = 5$ per group). Spermidine, green; amino acids, orange; glycerophospholipids, maroon; acylcarnitines, blue. Ornithine, an amino acid precursor for the polyamine pathway is indicated by a dotted circle. Metabolite concentrations and *p* values are provided in Supplemental Table S1. (C) Immunoblot of liver NNMT and γ tubulin. (D) Immunoblot of NNMT and γ tubulin in quadriceps. Data in panels C and D are quantified in Fig. 6D. (E) Kaplan-Meier survival curves of male mice (mean lifespan WT 109 weeks, $n = 21$, black; *Maf1*$^{-/-}$ 120 weeks, $n = 32$, red, $p = 0.24$ logrank test).

SUPPLEMENTAL TABLES

Table S1. Metabolite concentrations in plasma, skeletal muscle and liver. Concentrations ± SEM, fold change and *p* values are tabulated for the top scoring 31 metabolites in plasma, 28 metabolites in skeletal muscle and 22 metabolites in liver determined using the Biocrates

Absolute*IDQ* p180 system. Data are represented graphically in Fig. 4 and in Supplemental Figs. S4 and S6.

Table S2. RNA-seq analysis of wild-type and Maf1 KO eWAT.

This table contains raw and normalized RNA-seq read count data and EdgeR analysis of differential gene expression for reads that were uniquely mapped using HTseq-count. Data are represented graphically in Supplemental Fig. S5A.

Table S3. Pol III transcriptome RNA-seq scores.

This table contains RNA-seq scores for Pol III transcribed genes (see Materials and Methods). Data for precursor and mature tRNAs are represented graphically in Figs. 5A and 5B.

SUPPLEMENTAL MATERIALS AND METHODS

Animals, diets and analysis of lifespan

The *Maf1* targeting vector (Figure S1A) using for homologous recombination contained *loxP* sites in intron 1, 481 bp 5' of the start codon and in exon 8, 221 bp 3' of the stop codon. Chimeric mice were crossed to C57Bl/6 mice expressing Cre recombinase to obtain germ line transmission of the knockout allele. Wild-type and homogygous knockout mice were obtained by further breeding. The majority of the experiments were performed on animals obtained from homozygous crosses. Genotyping primers were Maf1 fwd: 5' AGG CTT GCA GGG CAG CAA TG 3', Maf1 WT rev: 5' CAC TGG CTG ACA GGG AGA TG 3' and Maf1 KO rev: 5' TGG CCC TTA GAG CTG GAG TG 3'. Mice were housed in barrier facilities at 22°C with a 12 h light/dark cycle and were fed *ad libitum* unless otherwise stated on a standard chow diet (KLIBA NAFAG #3436, 13% calories from fat), a breeder chow diet (PicoLab Rodent Diet 20, #5058, 21% calories from fat) or a high fat diet (Bio-Serv, 60% calories from fat). For pair-feeding, a

limiting amount of HFD (2.2 g per day) was determined for *Maf1*$^{-/-}$ mice from *ad libitum* feeding studies. Mice at 10 weeks of age that had been maintained on breeder chow were housed separately in standard cages and provided with pre-weighed high fat pellets every evening for eight weeks. Body weight was measured every three days. For lifespan studies, mice were maintained on breeder chow, *ad libitum,* until their natural end of life. A small number of animals were euthanized based on a veterinarian's independent assessment in accordance with AAALAC guidelines. These animals are represented in the lifespan analysis only if the condition of the animal was considered incompatible with continued survival.

A construct coding for a zinc finger nuclease targeting the *Maf1* gene, as well as corresponding mRNA, were obtained from Sigma-Aldrich (CompoZr®). Mice were produced by the Transgenic Mouse Facility of the University of Lausanne by injection of the zinc finger nuclease mRNA at a concentration of 40 ng/µl (Meyer et al. 2010). A total of 782 injected oocytes were transferred to 29 pseudopregnant females, of which 12 became pregnant and gave birth to 61 live pups. To detect founders, DNA was extracted from toe clips and mutations were identified by DNA sequencing with the primers Fwd 5'- ATG ACT CTG CCT GCG TTC TT-3' and Rev 5'-ACT CAT TGA GGG TGG CAA TC-3'.

Indirect calorimetry

Mice were housed individually at 22°C with a 12 h light/dark cycle and allowed to acclimate for 48 h before data collection. Gas exchange measurements were made under the following Oxymax system settings: air flow, 0.6 l/min; sample flow, 0.5 l/min; settling time, 55 sec; measuring time, 5 sec. Energy expenditure was calculated as recommended by the manufacturer using the following formulas. Heat = CV × VO2 and CV = 3.815 +1.232 × RER

where CV is the calorific value and RER is the respiratory exchange ratio. Calculations of energy expenditure were normalized for lean body mass.

Hyperinsulinemic-Euglycemic Clamp

The hyperinsulinemic-euglycemic clamp study was conducted over 180 min. in awake, freely moving mice following a 5 h fast. HPLC-purified [3-^{3}H]-glucose (NEN Life Sciences, Boston, MA) was prime-infused throughout the clamp [5 μCi bolus, followed by 0.05 μCi/min (basal) and 0.1 μCi/min (clamp)] to estimate the glucose disposal rate and hepatic glucose production. After an 80 min. basal period, a blood sample was collected from the tail tip for determination of basal glucose disposal rate (which equals basal hepatic glucose production in basal conditions). The clamp was initiated by prime-infusion of human insulin (Actrapid, Novo Nordisk, Denmark, 25mU/kg bolus, then 2.5 mU/kg/min), and 50% glucose was infused at variable rates and adjusted every 10 min. to clamp plasma glucose levels around 120 mg/dL as measured by glucometers on 2 μl blood samples (Ascensia Breeze2, Bayer Healthcare, Switzerland). After a 2 h stabilization period, blood was sampled from the tail tip 5 times at 10 minutes intervals for determination of glucose turnover under hyperinsulinemic, steady-state conditions. Hepatic glucose production (HGP) was measured as the difference between the Glucose Disposal Rate (GDR) anf the Glucose Infusion rate (GINF).

Western blotting and antibodies

Cell extracts (50 ug protein) were resolved by 7-11% SDS-PAGE and transferred to nitrocellulose membranes for antibody probing and detection with an Odyssey imager (LI-COR). Primary antibodies were obtained against HSL, phospho-HSL and LC3 (Cell Signaling Technology), UCP1 (Abcam ab47687), Nnmt (H-68, Santa Cruz Biotechnology), PLIN2, (OriGene Technologies, Inc.), α and γ tubulins (clone B-5-1-2 and GTU-88, Sigma-Aldrich),

GAPDH (GeneTex, GT239), and HADC (Abcam ab7030). Antibodies against full-length recombinant TBP were raised in rabbits. MitoProfile® Total OXPHOS Rodent WB Antibody Cocktail was from Abcam. Secondary antibodies were from LI-COR.

Mitochondrial-nuclear DNA ratio measurement.

Total DNA was isolated from the livers of 9 WT and 9 *Maf1*$^{-/-}$ (3 months old) mice under HFD with the Nucleospin kit from Macherey-Nagel. qPCRs were performed with equal amounts of DNA from each sample using SYBERGreen Master mix (Roche) and 5 µM of the following primers: mouse mitochondrial DNA measurements: Cyt B [forward, 5'-GTG AAC GAT TGC TAG GGC C-3'; reverse, 5'- CGA TTC TTC GCT TTC CAC TTC AT-3'], and ND1 [forward, 5'- CTC TTA TCC ACG CTT CCG TTA CG-3'; reverse, 5'-GAT GGT GGT ACT CCC GCT GTA-3']; mouse nuclear DNA measurements: H19 [forward, 5'-GTA CCC ACC TGT CGT CC-3'; reverse, 5'-GTC CAC GAG ACC AAT GAC TG-3']. Ratios of CytB:H19 and Nd1:H19 were calculated.

RNA-seq analysis of pol II and pol III transcriptomes and RT-qPCR

Three biologically independent samples (RNA integrity numbers >7.5, Agilent Bioanalyzer) from wild-type and *Maf1*$^{-/-}$ mice were sequenced. For analysis of the pol II transcriptome, uniquely mapped reads were aligned to the mouse genome (mm9) and counted using GSNAP and HTseq-counts, respectively (Wu and Nacu 2010;Anders et al. 2014). Normalization and statistical evaluation of differential gene expression was performed using EdgeR and DESeq (Robinson et al. 2010;Anders et al. 2013). In the analysis of the pol III transcriptome, sequence tags were mapped in three sequential steps. The first mapping was performed with Bowtie on the Mm9 Mouse genome assembly release. The tags left unmapped were then aligned with BLAT on the pol III loci listed in Table S3. As twenty four tRNA genes

contain introns, the remaining tags were aligned in a third round with BLAT to sequences corresponding to spliced tRNAs. The results of the three alignment steps were pooled and the tags aligning in the pol III loci listed in Table S3 were counted. The tags were scaled to the total number of tags aligning in pol II genes, and tags with multiple matches in the genome were given a weight corresponding to the number of matches divided by the number of times they were sequenced. Scores corresponding to the log2 of tags per gene were then calculated. Tags were considered derived from precursor tRNAs when they extended either upstream or downstream of the mature tRNA coding region or overlapped with tags extending upstream or downstream of the tRNA coding region or for tRNA genes containing an intron, when they contained intron sequences. Sequence tags were considered derived from mature tRNAs when they had 5' and 3' ends that mapped entirely within the mature RNA, and for tRNA genes containing an intron, when they spanned the exon-exon junction. For the differential analyses, two different approaches were used. In the first, we fitted the generalized linear model (GLM) from EdgeR(Robinson et al. 2010) on the scaled tag counts per gene for the three *Maf1*$^{-/-}$ versus the three WT samples; in the second, we applied the limma linear model fitting(Smyth 2004;Smyth et al. 2005) on the log2 of the scaled tag counts per gene, again for the three *Maf1*$^{-/-}$ versus the three WT samples. RT-qPCR was performed using SybrGreen detection as described previously (Reina et al. 2006). For the measurement of pre-rRNA, primers specific for the 5' external transcribed spacer were employed as reported previously (Oie et al. 2014). Differential gene expression was calculated using the ΔΔCt method using GAPDH as the internal reference.

Quantitation of total tRNA

Total RNA (5 μg) was resolved on denaturing polyacrylamide gels, stained with ethidium bromide, imaged and quantified using ImageQuant software. Amounts of total tRNA were

normalized to 5.8S rRNA. The intensity of tRNA and 5.8S rRNA staining was empirically determined to be in the linear range under these conditions.

Immunohistochemistry and fluorescence microscopy

Liver sections were stained for LC3B (Cell Signaling Technology, Inc., MA, USA) overnight at 4°C and incubated with Alexa Fluor® 647 Goat Anti-Rabbit secondary antibody for 1hr at room temperature (RT). For lipid droplet detection, sections were incubated with BODIPY 493/503 for 20 min. at RT. Images were acquired using a Leica DMI6000B microscope/DFC360FX 1.4-megapixel monochrome digital camera (Leica Microsystems, Germany) with a X100 objective/1.4 numerical aperture. All images were acquired at same exposure times. Image slices of 0.2μm thickness were captured and deconvolved using the Leica MetaMorph acquisition/analysis software. All images were prepared using Adobe Photoshop and subjected to identical post-acquisition brightness/contrast effects. Native images were processed with the "colocalization finder" plugin of the NIH ImageJ software, and areas of colocalization are shown as white pixels.

SUPPLEMENTAL REFERENCES

Anders S, McCarthy DJ, Chen Y, Okoniewski M, Smyth GK, Huber W, Robinson MD. 2013. Count-based differential expression analysis of RNA sequencing data using R and Bioconductor. *Nat Protoc.* **8**: 1765-1786.

Anders S, Pyl PT, Huber W. 2014. HTSeq - A Python framework to work with high-throughput sequencing data. *Bioinformatics.*

Meyer M, de Angelis MH, Wurst W, Kuhn R. 2010. Gene targeting by homologous recombination in mouse zygotes mediated by zinc-finger nucleases. *Proc.Natl.Acad.Sci.U.S.A* **107**: 15022-15026.

Oie S, Matsuzaki K, Yokoyama W, Tokunaga S, Waku T, Han SI, Iwasaki N, Mikogai A, Yasuzawa-Tanaka K, Kishimoto H et al. 2014. Hepatic rRNA transcription regulates high-fat-diet-induced obesity. *Cell Rep.* **7**: 807-820.

Reina JH, Azzouz TN, Hernandez N. 2006. Maf1, a new player in the regulation of human RNA polymerase III transcription. *PLoS One.* **1**: e134.

Robinson MD, McCarthy DJ, Smyth GK. 2010. edgeR: a Bioconductor package for differential expression analysis of digital gene expression data. *Bioinformatics.* **26**: 139-140.

Smyth GK. 2004. Linear models and empirical bayes methods for assessing differential expression in microarray experiments. *Stat.Appl.Genet Mol Biol* **3**: Article3.

Smyth GK, Michaud J, Scott HS. 2005. Use of within-array replicate spots for assessing differential expression in microarray experiments. *Bioinformatics* **21**: 2067-2075.

Wu TD, Nacu S. 2010. Fast and SNP-tolerant detection of complex variants and splicing in short reads. *Bioinformatics.* **26**: 873-881.

Bonhoure_Fig S1

A

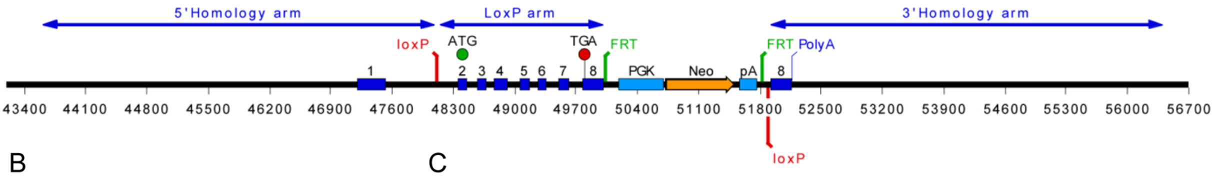

B

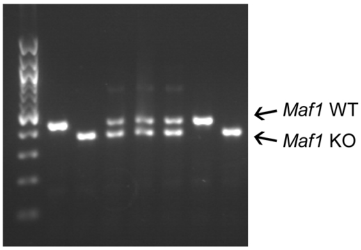

C

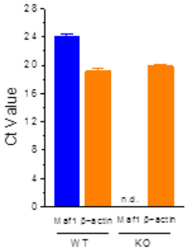

D

Genotypes of progeny from *Maf1*$^{+/-}$ crosses		
Maf1$^{+/-}$	*Maf1*$^{+/+}$	*Maf1*$^{-/-}$
148	79	56

Chi-squared test for independence p = 0.11

E

Genotype	Time between litters in homozygous crosses: Breeding pairs	Litters	Time between litters (days)	Litter size in homozygous crosses: Breeding pairs	Litters	Pups/litter
WT	18	76	25 ± 1	25	102	6.0 ± 0.2
Maf1$^{-/-}$	30	119	35 ± 1 *	39	165	4.8 ± 0.2 †

Values were calculated for breeding pairs having seven or fewer litters and report the mean ± s.e.m.
* p = 1.9 E-9, † p = 2.3 E-4.

F

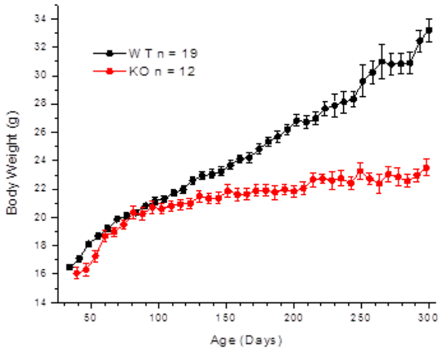

G

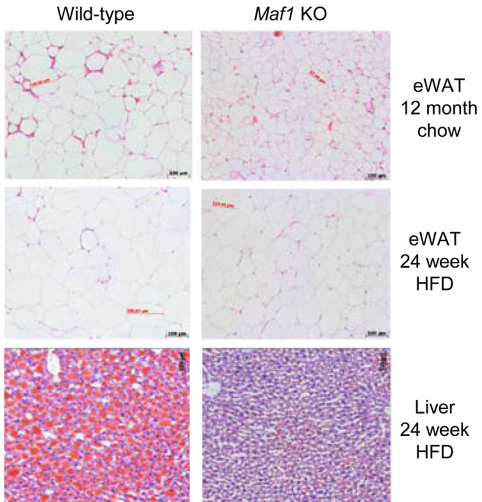

Bonhoure_Fig S2

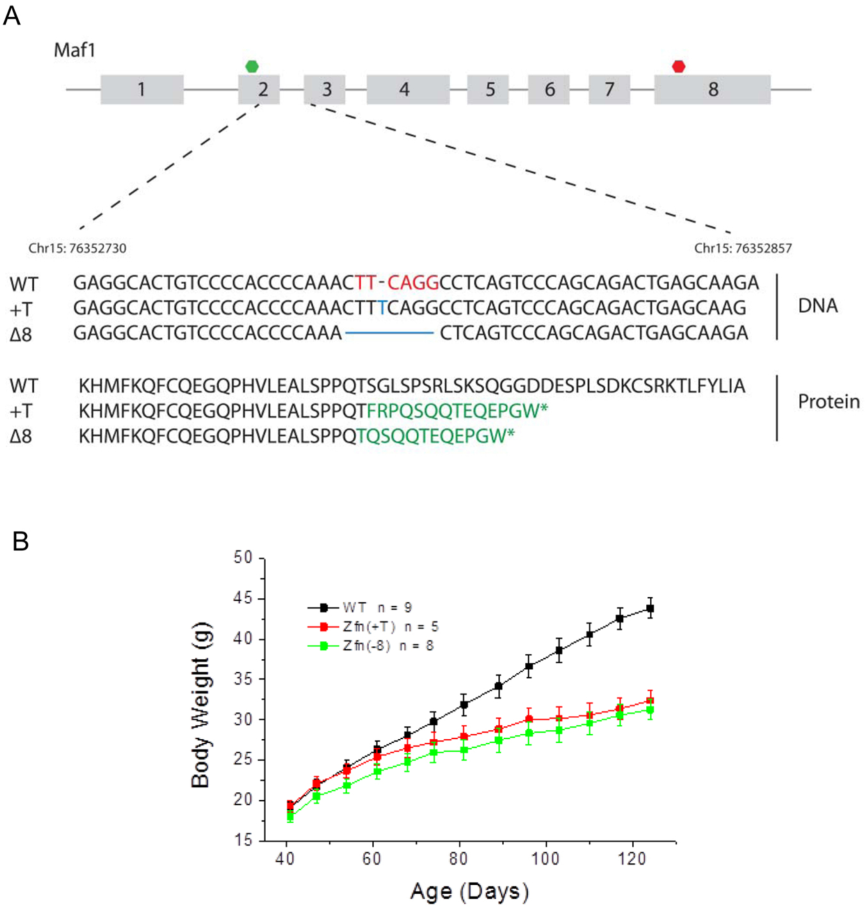

Bonhoure_Fig S3

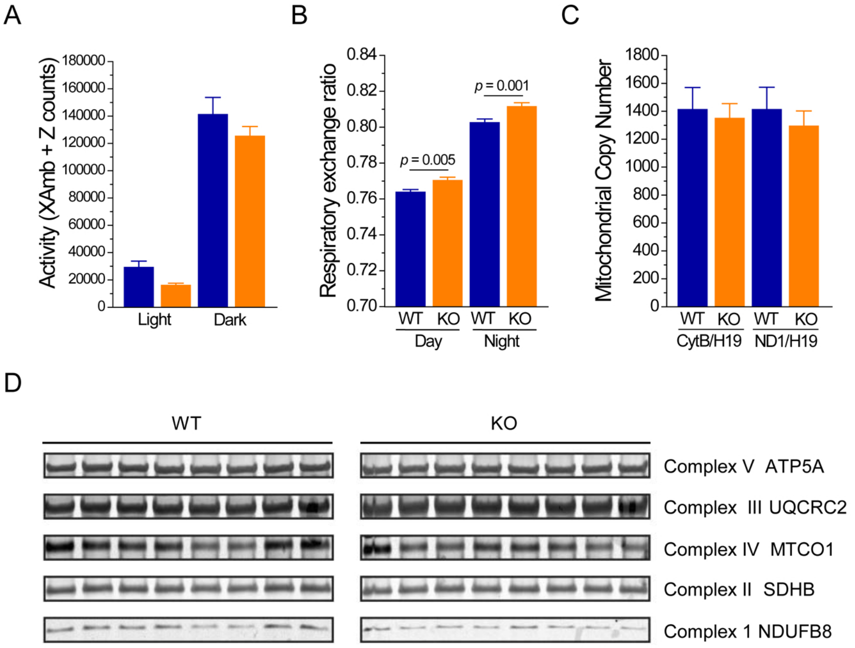

Bonhoure_Fig S4

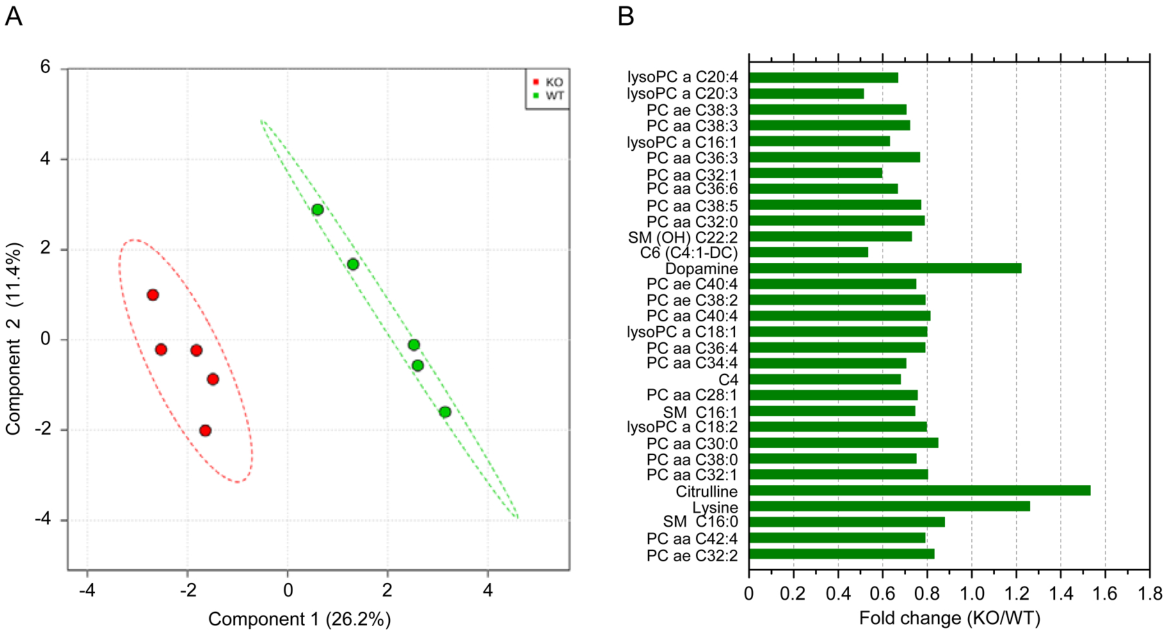

Bonhoure_Fig S5

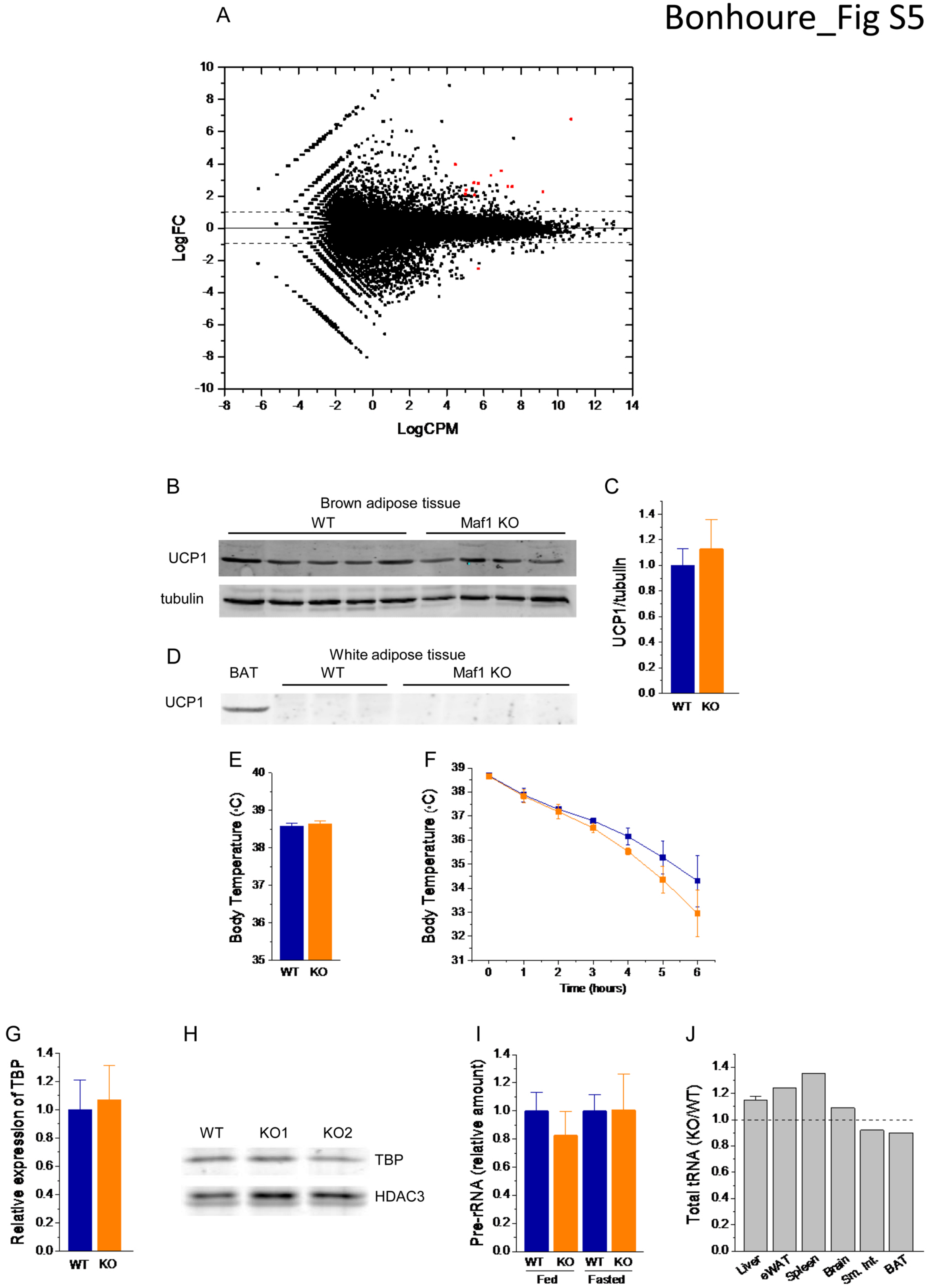

Bonhoure_Fig S6

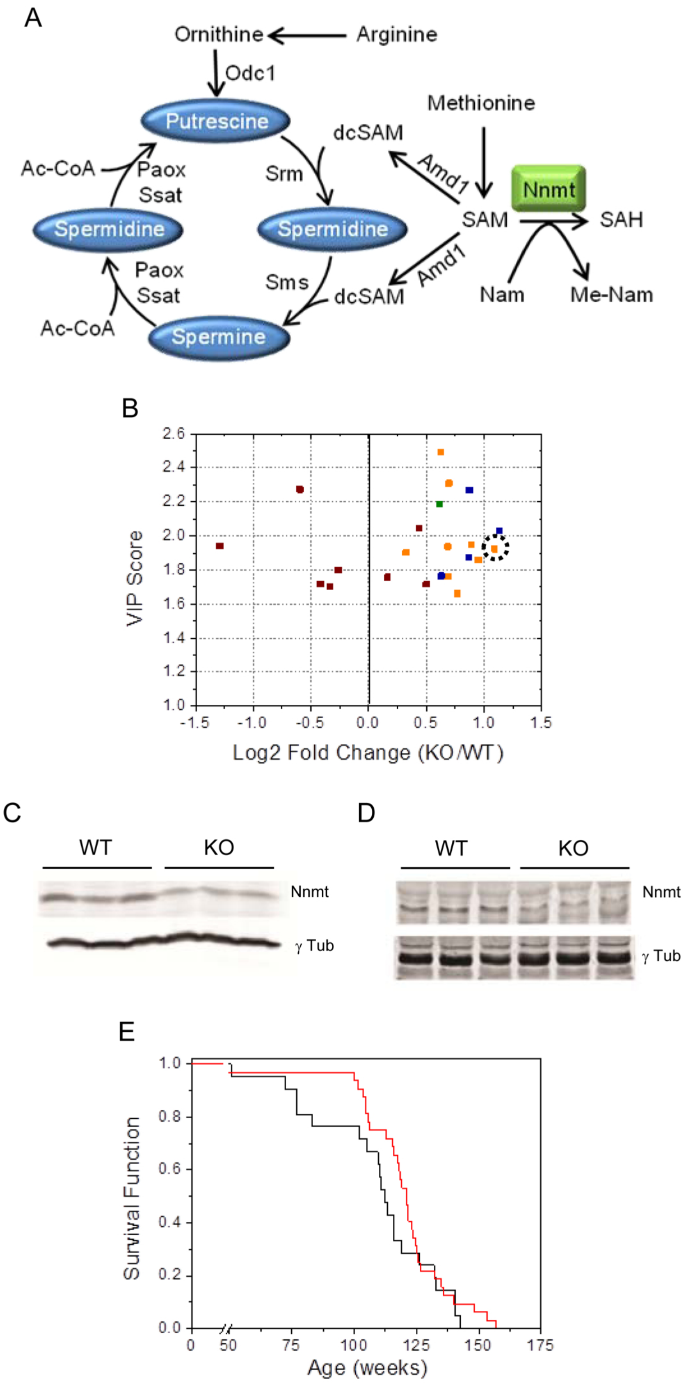

Supplement: Supplemental Material [file supp_29.9.934_SuppMaterial.pdf]
